# Disease-relevant β_2_-microglobulin variants share a common amyloid fold

**DOI:** 10.1038/s41467-023-36791-8

**Published:** 2023-03-02

**Authors:** Martin Wilkinson, Rodrigo U. Gallardo, Roberto Maya Martinez, Nicolas Guthertz, Masatomo So, Liam D. Aubrey, Sheena E. Radford, Neil A. Ranson

**Affiliations:** 1 Astbury Centre for Structural Molecular Biology, School of Molecular & Cellular Biology, Faculty of Biological Sciences, University of Leeds, Leeds, LS2 9JT UK; 2 Present Address: Aelin Therapeutics, Bio-Incubator Leuven, Gaston Geenslaan 1, 3001 Leuven, Belgium; 3 Present Address: Peak Proteins, Birchwood House, Larkwood Way, Macclesfield, Cheshire, SK10 2XR UK; 4 Present Address: Bicycle Therapeutics, Blocks A & B, Portway Building, Grant Park, Abingdon, Cambridge, CB21 6GS UK; 5 Present Address: Nara Medical University, 840 Shijo-cho, Kashihara, Nara, 634-8521 Japan

**Keywords:** Cryoelectron microscopy, Protein aggregation, Intrinsically disordered proteins

## Abstract

β_2_-microglobulin (β_2_m) and its truncated variant ΔΝ6 are co-deposited in amyloid fibrils in the joints, causing the disorder dialysis-related amyloidosis (DRA). Point mutations of β_2_m result in diseases with distinct pathologies. β_2_m-D76N causes a rare systemic amyloidosis with protein deposited in the viscera in the absence of renal failure, whilst β_2_m-V27M is associated with renal failure, with amyloid deposits forming predominantly in the tongue. Here we use cryoEM to determine the structures of fibrils formed from these variants under identical conditions in vitro. We show that each fibril sample is polymorphic, with diversity arising from a ‘lego-like’ assembly of a common amyloid building block. These results suggest a ‘many sequences, one amyloid fold’ paradigm in contrast with the recently reported ‘one sequence, many amyloid folds’ behaviour of intrinsically disordered proteins such as tau and Aβ.

## Abstract (web summary)

The authors use cryo-EM to determine amyloid fibrils structures of disease relevant variants of β2-microglobulin in vitro. Each variant is polymorphic, but all polymorphs from all samples are built from a a lego-like assembly of common building blocks, suggesting a one amyloid fold’ paradigm.

## Introduction

Self-assembly of peptides or proteins into amyloid fibrils is a complex process in which a protein precursor rearranges its initial structure and oligomerises to form elongated, unbranched fibrils with a cross-β fold. All amyloid fibrils have a common ordered core involving β-strands that are stacked parallel to the fibril axis and separated by ~4.8 Å^1,2^. Beyond this ordered core, disordered regions likely help define the fibril’s interactome and biological activity in vivo^3^. The deposition of amyloid fibrils is associated with an array of usually untreatable human diseases that are collectively known as amyloidoses. These include the well-known neurological disorders such as Alzheimer’s, Parkinson’s disease and amyotrophic lateral sclerosis (ALS), but also a range of systemic or localised amyloid disorders that affect organs including the heart, kidney, pancreas, kidney and others (for a review see^4^). Understanding the fundamental molecular mechanism(s) that underpin amyloid fibril formation is profoundly important, as it will provide the sequence-structure relationships needed to link aggregate form to disease, akin to structure-activity relationships in chemical reactions. Such an understanding should also reveal design principles to guide the use of amyloids as novel biomaterials^5^.

For many amyloid precursors (such as Aβ, tau and α-synuclein) the unit of aggregation is a peptide or protein that is wholly or substantially intrinsically disordered. However, a subset of precursors are stably folded, such as antibody light chains (LC), transthyretin (TTR) and β_2_-microglobulin (β_2_m)^4^. For this class of protein, transient unfolding/misfolding and complete or substantial reorganisation of their native structure is required for the cross-β structure of amyloid to form. The complex structural transformation from IDP or native fold into amyloid is under kinetic control and occurs on a rugged free-energy landscape. As a consequence, the mechanisms of assembly and the structure(s) of the fibrils that form depend on the conditions in which fibril growth occurs^6,7^. Sequence changes, such as mutation or post-translational modifications, can also change assembly mechanisms and can result in fibrils with a different architecture to wild-type^8,9^. This behaviour contrasts markedly with protein folding, in which the native structure of a protein is readily sought and found, even when substantial mutation to the polypeptide chain occurs^10^. In this context, it is not surprising that amyloid formation in vitro can result in multiple fibril forms, even when generated from an identical protein sequence: the concept of fibril polymorphism^11,12^. An increasing body of evidence suggests that this is not the case in vivo, where monomorphic fibril types of, e.g., tau^13–15^, Aβ^16^ and α-syn^17^ have been purified from brain tissue analysed post-mortem from donors with different neurological conditions, although fibril structure can depend on cell type^18^ and disease type^19^. This reinforces the concept that multiple fibril forms are kinetically accessible for each polypeptide sequence.

To better understand the molecular basis of fibril polymorphism, we here use cryoEM to determine the structure(s) of amyloid fibrils formed from three natural variants of β_2_m in vitro at physiological pH. Despite its discovery as the culprit protein of dialysis related amyloidosis (DRA) more than 35 years ago^20^, and being a paradigm for studies of amyloid formation mechanisms in vitro^21–25^, the structure of amyloid fibrils formed from native wild-type (wt)-β_2_m have not yet been elucidated. Native wt-β_2_m (99-residues in length) folds to an all anti-parallel immunoglobulin fold that is stabilised by a single disulphide bond (Cys25-Cys80)^26^. In individuals suffering from renal failure, β_2_m is no longer efficiently reabsorbed by the kidneys. As a consequence, its concentration in serum increases up to ~60-fold, which results in the formation and deposition of amyloid fibrils of wt-β_2_m and its truncated variant β_2_m-ΔN6 (along with other truncated forms^27^) in the joints. The resulting imbalance in osteoclast/osteoblast function caused by proteotoxicity associated with amyloid assembly, gives rise to a destructive joint arthropathy termed DRA^28,29^. By contrast with β_2_m-ΔN6 which forms amyloid fibrils rapidly at mildly acidic pH characteristic of arthritic joints (pH 6.2), wt-β_2_m does not readily aggregate into amyloid fibrils at this pH, unless co-solvents, cellular factors or other additives are included^30,31^. Indeed, our previous structure of the wt-β_2_m amyloid fibrils used samples assembled from the denatured state at pH 2.5 to overcome this problem^32^. Two naturally-occurring β_2_m variants involved in human amyloidosis have also been reported. The first of these, β_2_m-D76N was identified in 2012 as being responsible for a familial, systemic amyloidosis, wherein deposition of the variant occurs in the viscera in the absence of renal dysfunction^33^. The variant, β_2_m-V27M, was discovered more recently^34^. This variant is also associated with DRA, but its deposition is localised in the tongue and salivary glands, with some deposits also occurring in the joints.

The three variants, β_2_m-ΔN6, β_2_m-D76N and β_2_m-V27M, have different amyloid deposition sites and consequent diseases and occur with/without renal failure. Here we have characterised fibrils formed in vitro of all three variants using cryoEM, at a mildly acidic pH (pH 6.2) which mimics the arthritic joint environment and enables rapid and reproducible assembly of all three proteins into amyloid on a biochemically tractable timescale^35,36^. We show that the aggregation of each variant in vitro results in fibrils that are polymorphic, which contain different numbers of protofilaments and different numbers of β_2_m subunits per layer of the amyloid fibril structure. High resolution structures, however, show that this polymorphism arises from the assembly of a common building block that is strikingly homogeneous and monomorphic. Thus, mutations that affect pathology do not change the core folded amyloid conformation in vitro, despite displaying different final arrangements of these folds within their fibril architectures. These results suggest for β_2_m in vitro, a ‘many sequences, one amyloid fold’ paradigm is possible, in contrast with the ‘one sequence, many amyloid folds’ behaviour reported for intrinsically disordered proteins such as tau and Aβ in neurodegeneration.

## Results

### β_2_m variants form fibrils at different rates at near-neutral pH

**Fig. 1:**
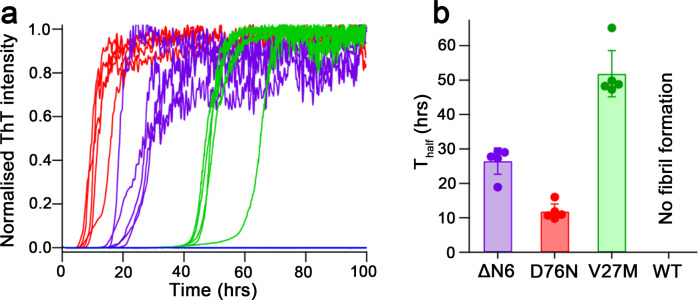
ThT kinetics of amyloid formation for the β_2_m variants at pH 6.2. **a** Normalised ThT fluorescence curves for WT-β_2_m (blue), β_2_m-∆N6 (purple), β_2_m-D76N (red) and β_2_m-V27M (green) at pH 6.2, showing five repeats (*n* = 5 technical replicates) for each protein. **b** Bar chart of the measured mean T_half_ values and (*n* = 5 technical replicates, using different batches of the protein). The error bars indicate standard deviation from the individual ThT runs (shown as filled spheres)), indicating the relative rate of amyloid formation for each protein. WT-β_2_m does not form amyloid at pH 6.2 during the 100 h reactions. Source data are provided as a Source Data file.

For this study, β_2_m-∆N6, β_2_m-D76N and β_2_m-V27M fibrils were grown from a native folded state, with aggregation reactions initiated in the presence of ThT and grown at 37 °C with constant shaking, in a buffer of 25 mM Sodium phosphate pH 6.2, 115 mM NaCl (Fig. 1a). Under these conditions, WT-β_2_m does not show an increase in ThT fluorescence over the 100 h reaction timescale, whilst all of the pathologically-relevant β_2_m variants readily aggregate at this pH and formed fibrils. Indeed WT-β_2_m only assembles into amyloid in vitro in acidic pH growth conditions, explaining why we previously determined the cryoEM structure of WT-β_2_m fibrils grown at pH 2.5^32^. β_2_m-D76N forms amyloid the fastest under these conditions (T_half_ of 12 ± 2 h), twice as fast as β_2_m-∆N6 (26 ± 3 h), which is in turn twice as fast as β_2_m-V27M (51 ± 7 h) (Fig. 1b). To investigate and compare the structure of the fibrils formed by each of the variants, we assembled fibrils of each protein in identical buffer conditions to the plate reader assay, but in Eppendorf tubes without ThT. After a few weeks, each sample had formed fibrils suitable for structure determination by cryoEM.

### Structure and polymorphism of β_2_m-ΔN6 amyloid fibrils

**Fig. 2:**
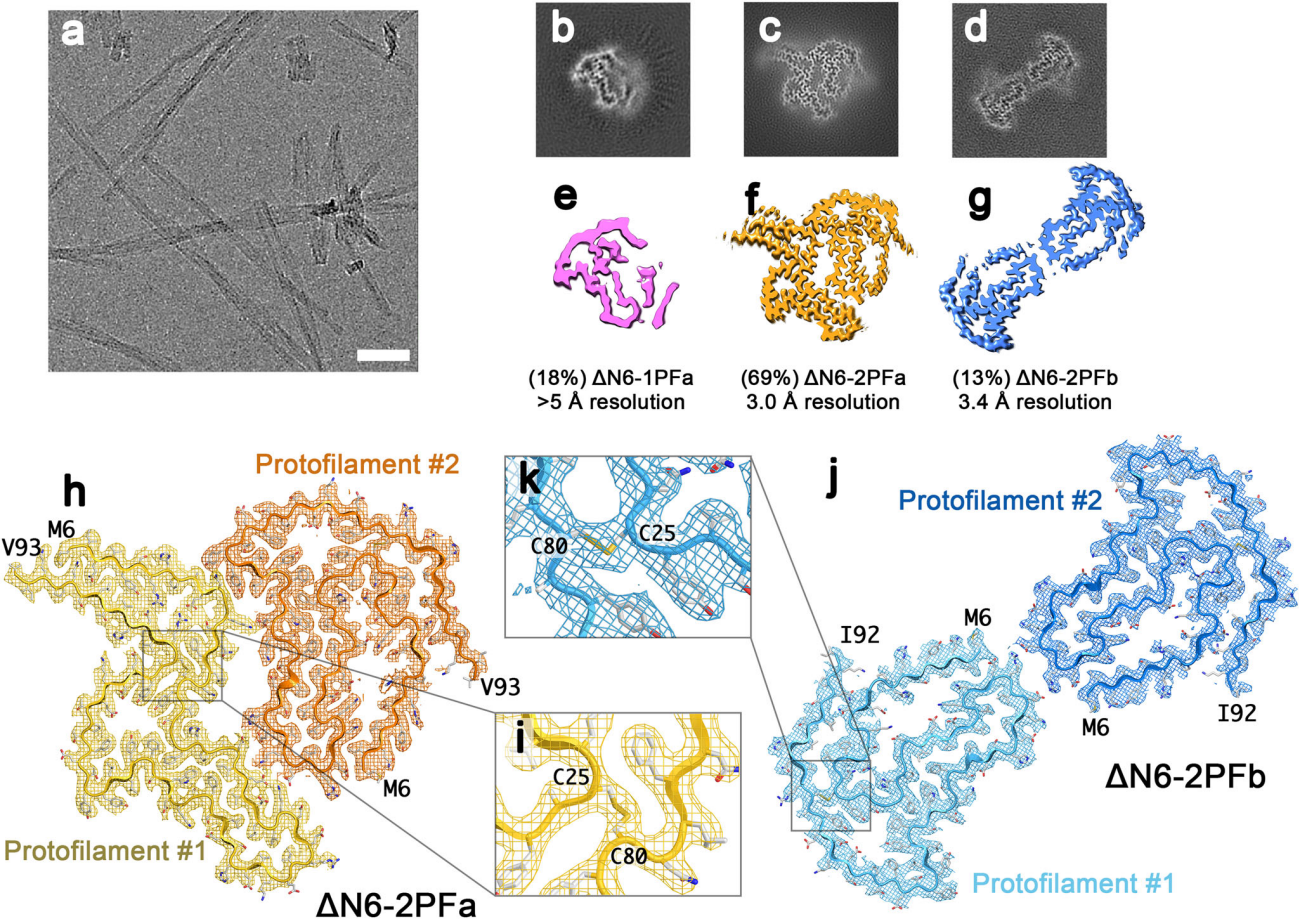
Polymorphism in β_2_m-ΔN6 fibrils. **a** Representative cryoEM micrograph of β_2_m-ΔN6 fibrils (scale bar corresponds to 50 nm). Two independent experiments were performed, with similar results. **b**–**d** Cross sections through the 3D reconstructions of the three polymorphs found in the β_2_m-ΔN6 data: **b** ΔN6-1PFa, **c** ΔN6-2PFa and **d** ΔN6-2PFb. For each map, a sum of the reconstructed densities for six XY-slices is shown, corresponding to one ~4.8 Å repeat of the amyloid core of the fibril. **e**–**g** Surface representation of the same structures with relative distributions and final map resolutions annotated. **h**, Refined density at 3.0 Å resolution for the ΔN6-2PFa structure with its accompanying atomic model. Protofilament #1 is in yellow, and protofilament #2 is orange. The EM density is shown as a mesh, and the atomic model as a cartoon with the same colour, and sidechains coloured according to a CPK scheme. **i** The disulphide bond between residues 25 and 80 is present and well-resolved. **j**, **k**, as for **h**, **i**, but showing the 3.4 Å structure of ΔN6-2PFb and the accompanying atomic model, with protofilament #1 in light blue, and protofilament #2 in darker blue.

Recombinant β_2_m-ΔN6 was used to form amyloid fibrils under conditions identical to those employed previously to determine its mechanism of fibril assembly (pH 6.2)^36,37^ (Methods). These studies demonstrated a defined reaction mechanism for β_2_m-ΔN6 fibril assembly involving the formation of specific dimers and hexamers that retain an Ig fold, but the conformational transition to the final fibril form remained elusive^36^. Fibril formation was monitored over time using negative-stain electron microscopy (nsEM) and a cryoEM dataset was collected when sufficient fibrils had formed (Fig. 2a; Methods). Initial 2D classification revealed that the sample is polymorphic, containing both narrow and wide fibrils (Supplementary Fig. 1). Subsequent 3D classification revealed that the narrow fibrils are built from a single protofilament (1PF) with one β_2_m molecule per layer, while wide fibrils contain two protofilaments (2PF) (Fig. 2b–g). Further 3D classification allowed the 2PF fibrils to be separated into two sub-types (Supplementary Fig. 1). We refined the 1PF fibril (ΔN6-1PFa; Fig. 2b, e) although we were unable to solve the structure to near atomic resolution given the rarity of this polymorph (~18% (Supplementary Fig. 1) (final resolution ~5 Å)). Most of the dataset (82% of all data) contained 2PF fibrils. The majority of these (69% of all data) describe a side-by-side 2PF arrangement, termed ΔΝ6-2PFa, which we were able to solve to 3.0 Å resolution (Fig. 2c, f, h, i). A minority of the wider fibrils (13% of all data) are in a different conformation, in which the two protofilaments are arranged ‘tail-to-tail’. We dub this form ΔN6-2PFb, and were able to solve its structure to 3.4 Å resolution (Fig. 2d, g, j, k). All three polymorphs (indeed all structures studied herein) have a left-handed helical twist, as judged by atomic force microscopy (AFM) (Supplementary Fig. 2a). Moreover, AFM height distribution analysis shows that the heights/widths of fibrils measured by AFM^38^ are consistent with the three polymorphs identified in the cryoEM dataset (Supplementary Fig. 2b).

**Fig. 3:**
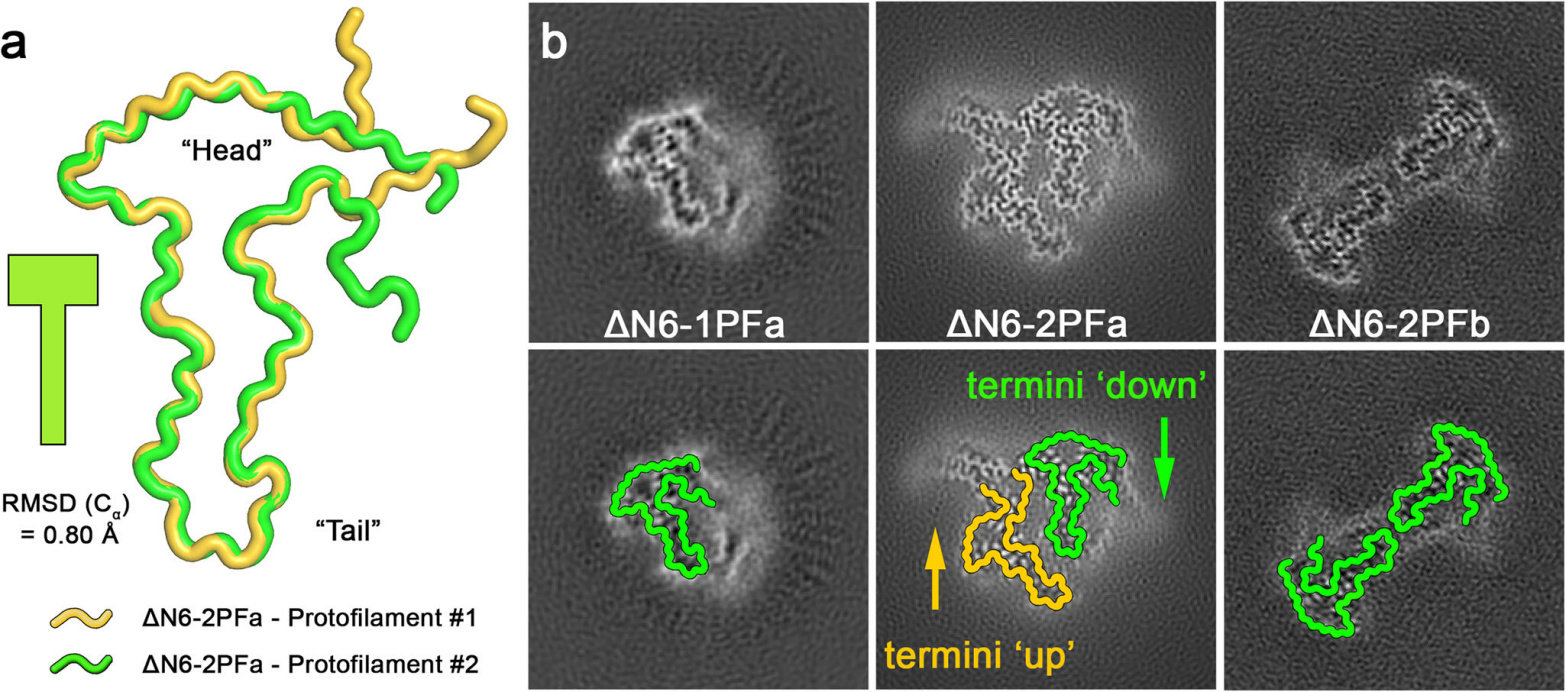
A hammer-shaped motif makes up the core of all β_2_m-ΔN6 polymorphs. **a** The backbone residues for the atomic model of ΔΝ6-2PFa was used to create tube representations of protofilament #1 (yellow) and protofilament #2 (green), defining schematics that contain the hammer shaped motif and indicating the direction the termini will pack. **b** Overlay of the termini-up schematic from protofilament #1 (yellow) and the termini down schematic from protofilament #2 (green) on the three polymorphs identified in the β_2_m-ΔN6 dataset: 1PFa, 2PFa and 2PFb, as indicated.

The density for ΔΝ6-2PFa was of sufficient quality to allow an unambiguous atomic model to be built for residues 6–93 of the ΔN6 chain (which constitutes residues 7–99 of the wt-β_2_m sequence) (Fig. 2h). This model shows that the amyloid core of the β_2_m subunit is a hammer-shaped fold that is delineated by the single intramolecular disulphide bond (Cys25-Cys80) for which we see clearly resolved density in the structure (Figs. 2i, 3a,b). The same motif is found in both protofilaments of the ΔΝ6-2PFa structure, but the two protofilaments differ in the arrangement of their N- and C-terminal regions, i.e. those residues that lie outside residues 25–80 in the sequence. In protofilament #1, the termini are oriented ‘upwards’, i.e., away from the hammer motif, whilst in protofilament #2, the termini are oriented ‘downwards’, i.e., they fold back and pack alongside the hammer motif of the originating subunit. The upward orientation of the termini in protofilament #1 exposes a polar and charged surface that protofilament #2 can bind against, permitting the side-by-side arrangement observed. An initial inspection of the cryoEM density for the second 2PF fibril (ΔN6-2PFb) polymorph revealed that the same hammer motif defined by the intramolecular disulphide bond is observed in each protofilament of this structure. However, this polymorph has C2 symmetry, and the two subunits in each molecular layer are thus identical. Using our atomic model for ΔΝ6-2PFa, we built a model for the 2PFb subunit that encompasses residues 6–92. Notably in this structure the N- and C-termini of each subunit adopt a ‘downwards’ conformation. This blocks the inter-protofilament surface observed in ΔN6-2PFa. Instead, the two protofilaments in ΔN6-2PFb dock via electrostatic interactions in the base of the ‘handle’ region of each protofilament (Fig. 2j).

The three different polymorphs of β_2_m-ΔN6 observed thus share a common, hammer-shaped amyloid fold involving residues 25–80, that can assemble in different ways to create the three fibril structures observed, which differ only in the orientation of the ‘tails’ formed from the visible 19 and 13 residues N- and C-terminal to the disulphide bond, respectively (Fig. 3a, b). To illustrate this, we derived a backbone cartoon for residues 15–85 from each protofilament in the highest resolution structure obtained (ΔN6-2PFa), and coloured these yellow for the ‘termini up’ conformation (as found in protofilament #1) and green for the ‘termini down’ conformation (as found in protofilament #2) (Fig. 3a). Each molecular layer in all three polymorphs, including the single protofilament structure (ΔN6-1PFa), is built from one of these two related building blocks. The results thus portray fibril polymorphism generated by the different docking of protofilaments with a common structural fold, with diversity in the fibrils assembled generated by differences in the orientation of the termini of the interacting protofilaments.

### Structure and polymorphism in β_2_m-D76N amyloid fibrils

Amyloid formation of the variant β_2_m sequence, β_2_m-D76N, which bears a single Asp to Asn substitution in a solvent exposed loop of the native monomer was next investigated. Previous work has shown that this single amino acid substitution results in rapid aggregation of the variant protein into amyloid fibrils in vitro at neutral and mildly acidic pH 6.2, while wt-β_2_m is aggregation resilient^35,39–43^ (Fig. 1). Despite substantial research effort^44^, however, including recent design and selection of >60 variants^45^, why this single amino acid substitution (but not similar single substitutions elsewhere in the protein) has such a dramatic effect on the amyloid potential of the protein remains unclear. Patients bearing the β_2_m-D76N mutation are heterozygous, and the serum concentration of β_2_m is not increased^33^. Nevertheless, full-length β_2_m-D76N is deposited throughout the visceral organs, establishing the D76N variant as being responsible for a familial, systemic amyloidosis (for a review see ref. ^21^).

**Fig. 4:**
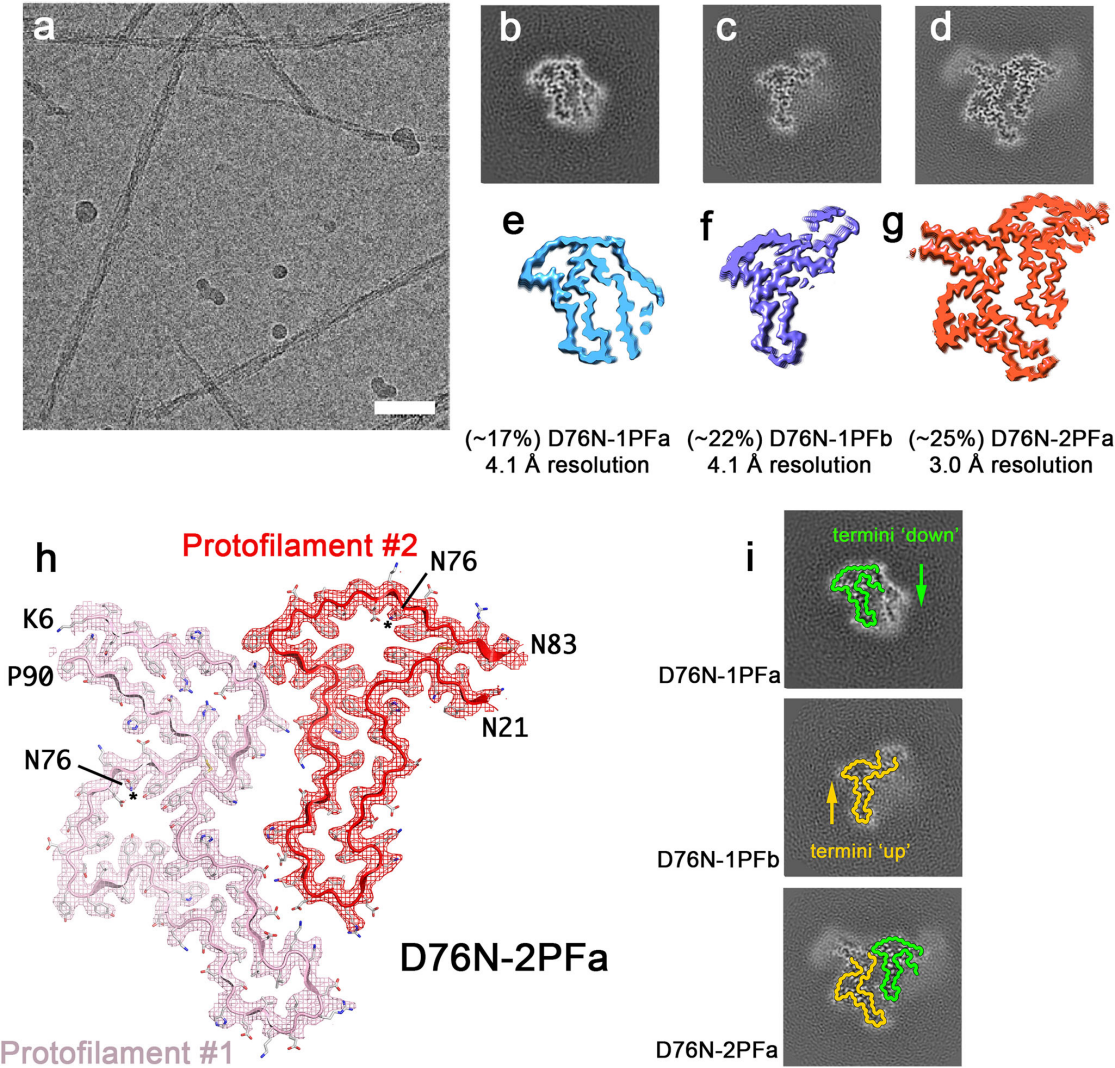
Polymorphism in β_2_m-D76N fibrils. **a** Representative cryoEM micrograph of β_2_m-D76N fibrils (scale bar corresponds to 50 nm). Two independent experiments were performed, with similar results. **b**–**d** Cross sections through the 3D reconstructions of the three polymorphs found in the β_2_m-D76N data: **b** D76N-1PFa, **c** D76N-1PFb and **d** D76N-2PFa. As in Fig. 2, cross-section represents the sum of the reconstructed densities for six XY-slices, equivalent to one rung of the amyloid fibril. **e**–**g** Surface representation of the same structures with relative distributions and final map resolutions annotated. **h** Refined density at 3.0 Å resolution for the D76N-2PFa structure with its accompanying atomic model. Protofilament #1 is in pink, and protofilament #2 is red. The EM density is shown as a mesh, and the atomic model as a cartoon with the same colour, and sidechains coloured according to a CPK scheme. **i** Overlay of the β_2_m-ΔN6 fibril schematics from Fig. 2a on the β_2_m-D76N fibril polymorphs.

To investigate how the single amino acid substitution, D76N, affects the structure of fibrils formed, we again initiated amyloid assembly reactions for recombinant β_2_m-D76N at pH 6.2 and monitored formation of amyloid fibrils by nsEM. A cryoEM dataset was again taken when fibrils had formed (Fig. 4a; Methods). Initial 2D classification revealed that the sample was more polymorphic than fibrils formed from β_2_m-ΔΝ6, but again contained both narrow and wide fibrils (Supplementary Fig. 3) consistent with height analysis from AFM images (Supplementary Fig. 2b). Subsequent 3D classifications revealed that a mixture of 1PF and 2PF forms were present, which allowed us to refine the structures of three distinct fibril polymorphs: two different 1PF filaments and a single 2PF form (Fig. 4b–g). These forms constitute ~65% of the fibril segments imaged, with the remaining particles separating into classes that did not refine to high resolution, perhaps reflecting a lower degree of order in the fibrils overall (Supplementary Fig. 3).

Again, we were not able to solve the structure of the 1PF fibrils to near atomic resolution and so did not build atomic models for these polymorphs. The cryoEM density for D76N-2PFa, however, was of sufficient quality to allow an unambiguous atomic model to be built, although the amount of resolved protein sequence per layer is lower than for the equivalent structures of fibrils of β_2_m-ΔN6 (i.e. ΔN6-2PFa) (Fig. 4h). Strikingly, protofilament #1 has a similar structure to protofilament #1 in ΔΝ6-2PFa with residues 6 to 90 resolved (Fig. 4h). The length of ordered polypeptide chain resolved in protofilament #2 is shorter, with only residues 21–83 being sufficiently ordered to build a model. This is consistent with the notion that β_2_m-D76N fibrils are less ordered than those of β_2_m-ΔΝ6. Despite this difference, the same hammer shaped motif is apparent (Fig. 4i). The cartoon traces used in Fig. 3 to describe all of the observed fibrils of β_2_m-∆N6 describe the β_2_m-D76N fibril polymorphs equally well (Fig. 4i), regardless of whether they contain one or two protofilaments. Thus, we again observe fibril polymorphism that is defined by the different assembly of a common core amyloid fold. Single protofilament fibrils are observed with either the ‘termini down’ (D76N-1PFa) or ‘termini up’ (D76N-1PFb) subunit conformations, and these come together to form the 2PFa fibril.

### Structure and polymorphism in β_2_m-V27M amyloid fibrils

**Fig. 5:**
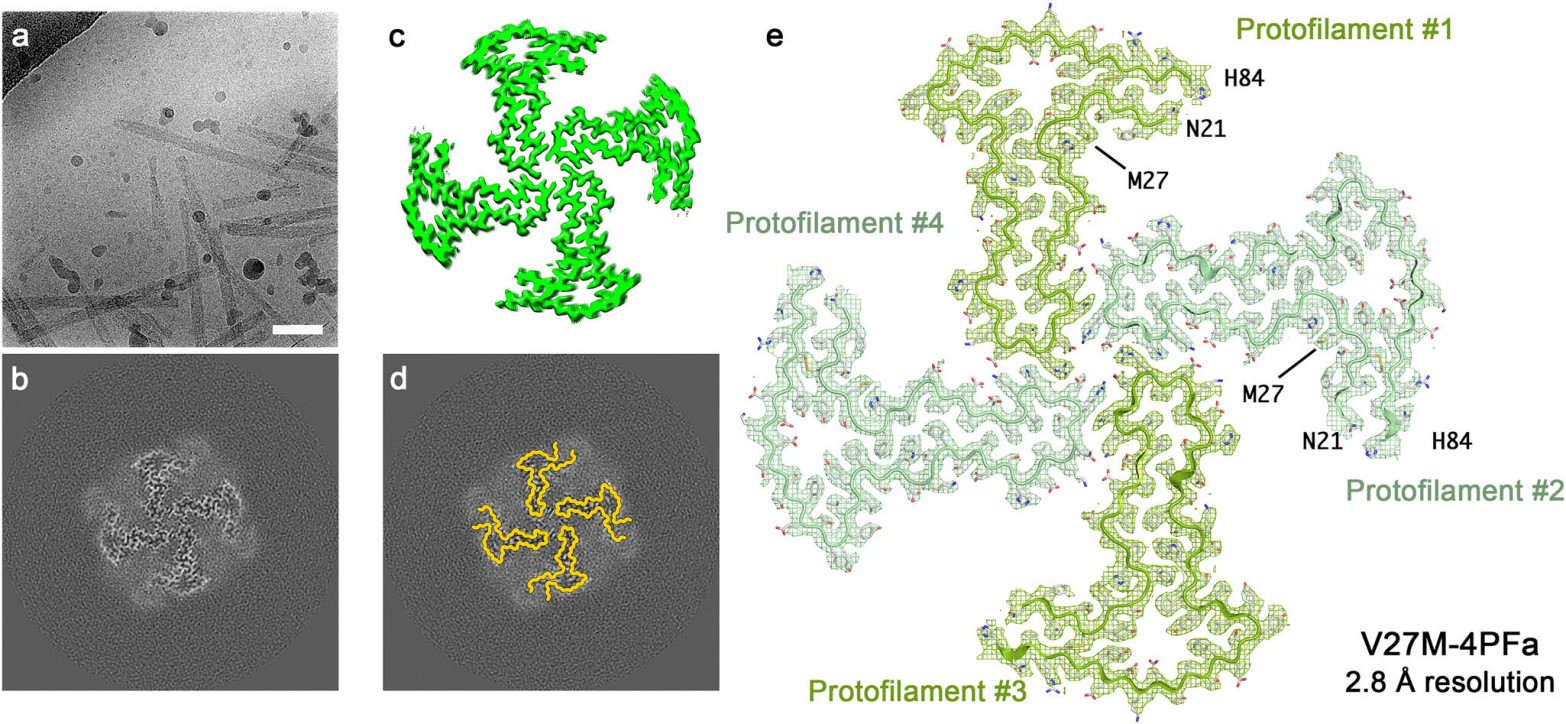
Polymorphism in β_2_m-V27M fibrils. **a** Raw cryoEM micrograph of β_2_m-V27M fibrils (scale bar corresponds to 50 nm). Two independent experiments were performed, with similar results. **b** Cross-section through the 3D reconstruction of the four protofilament polymorph found in the β_2_m-V27M dataset that could be processed to high resolution. As in Figs. 2 and 4, this cross-section represents the sum of the reconstructed densities for six XY-slices, equivalent to one rung of the amyloid fibril. **c** Surface representation of the same structure in cross section. **d** Overlay of the β_2_m-ΔN6 schematics from Fig. 2a on the β_2_m-V27M 4PF fibril polymorph. All four subunits per molecular layer conform to the ‘termini-up’ conformation. **e** Refined density at 2.8 Å resolution for the V27M-4PFa fibril structure with its accompanying atomic model. Protofilaments #1 and #3 are in olive green, and protofilaments #2 and #4 are in green. The EM density is shown as a mesh, and the atomic model as a cartoon with the same colour, and sidechains coloured according to a CPK scheme.

Recent studies have identified a second naturally-occurring β_2_m sequence variant, V27M, that is associated with amyloid disease^34^. β_2_m-V27M amyloid deposits were discovered in the tongue and salivary glands of a patient living with long-term haemodialysis, by contrast with the deposition of amyloid in joints characteristic of wt-β_2_m and β_2_m-ΔN6 in DRA. To examine how the substitution of a Val to Met at residue 27, which is buried in the core of native β_2_m and located close to its disulphide bond, alters the structure or polymorphism of the amyloid fibrils that result from its self-assembly, we again initiated amyloid assembly at pH 6.2 (Methods). CryoEM images revealed that the gross morphology of β_2_m-V27M fibrils is very different to those of β_2_m-ΔN6 and β_2_m-D76N fibrils, with the dominant fibril form being thicker and less overtly twisted (Fig. 5a). This was confirmed by 2D classification, although narrower fibrils are also present (Supplementary Fig. 4). Subsequent 3D classifications revealed that 69% of the fibril segments selected correspond to a new polymorph containing four protofilaments (i.e. four β_2_m-V27M monomers per layer of the amyloid structure) (Fig. 5b, c). We term this polymorph V27M-4PFa, and were able to refine its structure to 2.8 Å resolution. This revealed fibrils with a propeller-like appearance in cross-section, an organisation of protofilaments unseen for any other amyloid structure to date^12^. The remainder of the β_2_m-V27M fibril dataset contains narrower fibrils, presumably containing one or two protofilaments, but we were unable to refine these to high resolution, suggesting variable/disordered conformations. AFM height distribution analysis of β_2_m-V27M fibrils showed fibril widths consistent with the presence of 1PF, 2PF and 4PF species (Supplementary Fig. 2b). A greater proportion of narrower fibrils were seen by AFM imaging (~60% with <8 nm fibril height) than in the cryoEM dataset, potentially due to the different sample deposition environments between the techniques. Despite this, a cross-sectional area calculated from one of the wider V27M fibrils strongly resembled the 4PFa fibril structure determined by cryoEM (Supplementary Fig. 5), suggesting that this same species is present, albeit with different relative abundances.

Examination of the cryoEM density for V27M-4PFa revealed that, despite the unique morphology of this fibril form, the β_2_m-V27M protein chains adopt a ‘termini-up’ hammer motif similar to that observed in both the β_2_m-∆N6 and β_2_m-D76N fibril samples. This conformation is present in each of the four protofilaments in the V27M-4PFa structure. To illustrate this, a backbone cartoon for a ‘termini up’ subunit generated from the ΔΝ6-2PFa structure (Fig. 3a) was overlaid on each of the four protofilaments of β_2_m-V27M (Fig. 5d). The cryoEM density was of sufficient quality to build an atomic model for residues 21–84 of each chain (Fig. 5e). The fibril has C2 symmetry, with all four subunits per molecular layer adopting a near-identical conformation, but is packed as a dimer of dimers, with the dimer formed from protofilaments #1 and #2 being identical to that formed by protofilaments #3 and #4 (Supplementary Fig. 4).

### The hammer motif is the common building block of β_2_m amyloid polymorphs

**Fig. 6:**
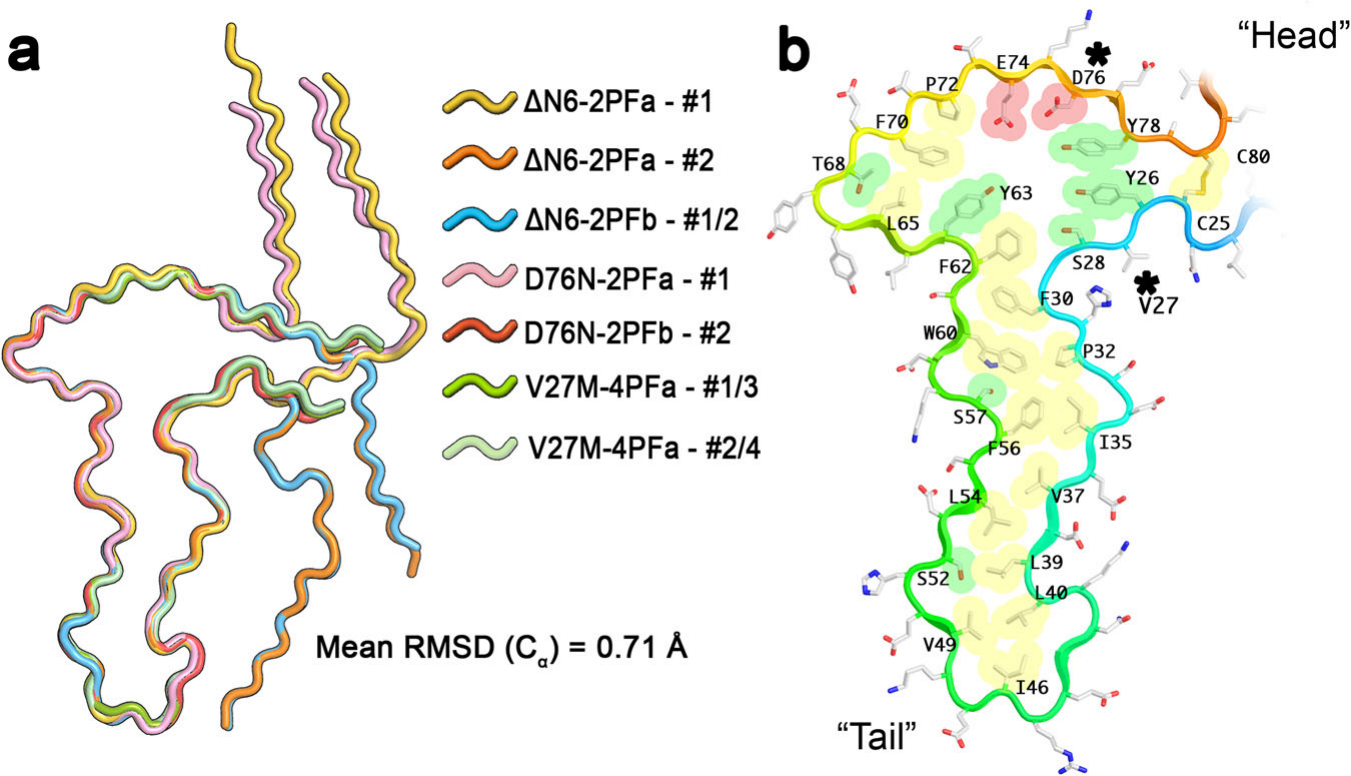
The hammer shaped motif is identical in all fibril structures solved. **a** Overlay of the seven unique chains in the atomic models built for the β_2_m polymorphs described above. The reference was protofilament #1 from β_2_m-ΔN6-2PFa, and each unique chain was aligned to the reference across the Cα atoms for residues 25–80 inclusive. **b** The hammer-shaped amyloid motif. Again, protofilament #1 from β_2_m-ΔN6-2PFa is shown, with the backbone cartoon coloured in a spectrum from blue (N-terminus) to red (C-terminus), and with residues labelled. Side chains projecting into the amyloid core are coloured according to their chemical character: hydrophobic = yellow, polar = green and acidic=red).

Combining information from all the β_2_m amyloid structures determined here, three types of polymorph are observed: single protofilaments (for β_2_m-ΔΝ6 and β_2_m-D76N), side-by-side two protofilament forms (β_2_m-ΔΝ6 and β_2_m-D76N), and tail-to-tail protofilaments (β_2_m-∆N6 and β_2_m-V27M). A comparison of the subunits across the different polymorphs confirms that all are constructed from assemblies of a common core motif; superposition of residues 25–80 of the seven unique chains from our structure determinations (i.e. ΔΝ6-2PFa (protofilaments #1 and #2), ΔN6-2PFb (protofilament #1), D76N-2PFa (protofilaments #1 and #2) and V27M-4PFa (protofilaments #1 and #2)) are shown in Fig. 6a. Taking ΔΝ6-2PFa PF1 as the reference, the average pairwise RMSD between the Cα atoms for residues 25–80 is 0.71 Å, with a maximum individual score of 0.82 Å. The fold of each protofilament in each structure is not only similar in cross section, but they also share a similar path in their ‘Z-height’, with each molecular layer contacting three others (Supplementary Fig. 6). This demonstrates that the hammer-shaped core motif in each amyloid fibril is identical at the resolution of the structures determined.

The amyloid hammer motif is delimited by the disulphide bond between residues 25 and 80, which is present in the native β_2_m fold. In contrast to the parallel orientation of the β-strands that link residues C25 and C80 in the native β_2_m fold, these segments are anti-parallel in the amyloid state (Fig. 6a). Given that the disulphide bond must be intact for fibril formation in vitro and in vivo^46,47^, β_2_m must undergo a substantial unfolding event to achieve the amyloid state, consistent with observations for LC amyloid formation^48–51^ Importantly, for all amyloid structures determined here, P32 is in a non-native *trans* conformation, consistent with previous studies which indicated that *cis* to *trans* isomerization of P32 plays a central role both in folding to the native immunoglobulin domain structure and in amyloid formation^23–25,52–54^.

The intramolecular interactions which stabilise the hammer-like motif are largely hydrophobic (Fig. 5b). These include the recognised amyloid promoting region (APR; residues 60–66) of β_2_m^45^, although the APR represents only a small proportion of the stabilising interactions within the fibril core. The vast majority of the charged and polar residues have side chains that project into solvent, or are placed such that they can interact with other protofilaments to form higher order structures (Fig. 5b). A notable area of exception to this however, is within the head of the hammer where a cluster of polar residues point towards an internal pocket within the fold. The resolution of the map is insufficient to identify ordered solvent, but the pocket does contain weak density that could be occupied by partially ordered solvent molecules (Supplementary Fig. 7). The amyloid core also contains the sites of both the D76N and V27M amino acid substitutions. Notably, Val/Met27 projects into solvent, while Asp/Asn76 projects into the core of the amyloid fold, where it is stabilised by interactions with adjacent polar residues within the hammer-head (Fig. 6b). Hence, both substitutions (asterisks in Fig. 6b) are readily accommodated by the core amyloid fold. Superposition of the area surrounding each mutation with that of the ∆N6 fibril model (which has the WT sequence in these regions) shows that the hammer fold is not significantly altered by either mutation (Supplementary Fig. 8). There are, however, implications for the conformation of the chain N-terminal and C-terminal to the disulphide bridge. For D76N, this is a subtle constriction of the termini towards the hammer fold by ~3 Å (Supplementary Fig. 8a). For V27M, a potential steric block of the substituted methionine with N21 would potentially disfavour the ‘termini down’ conformation (Supplementary Fig. 8c). This position however is only completely ordered for ∆N6 protein chains and the partial ‘termini down’ D76N 2PFa protofilament #1 conformation is in fact the closest matching structure to that of the V27M protein chains (RMSD of 0.35 Å over 56 Ca atoms, Supplementary Fig. 8d). It is not therefore possible to discern why the V27M mutation favours the novel 4PF propellor assembly over the 2PF fibril polymorphs seen for the other variants.

### Side-by-side fibril polymorphs are built using polar and charged interactions

**Fig. 7:**
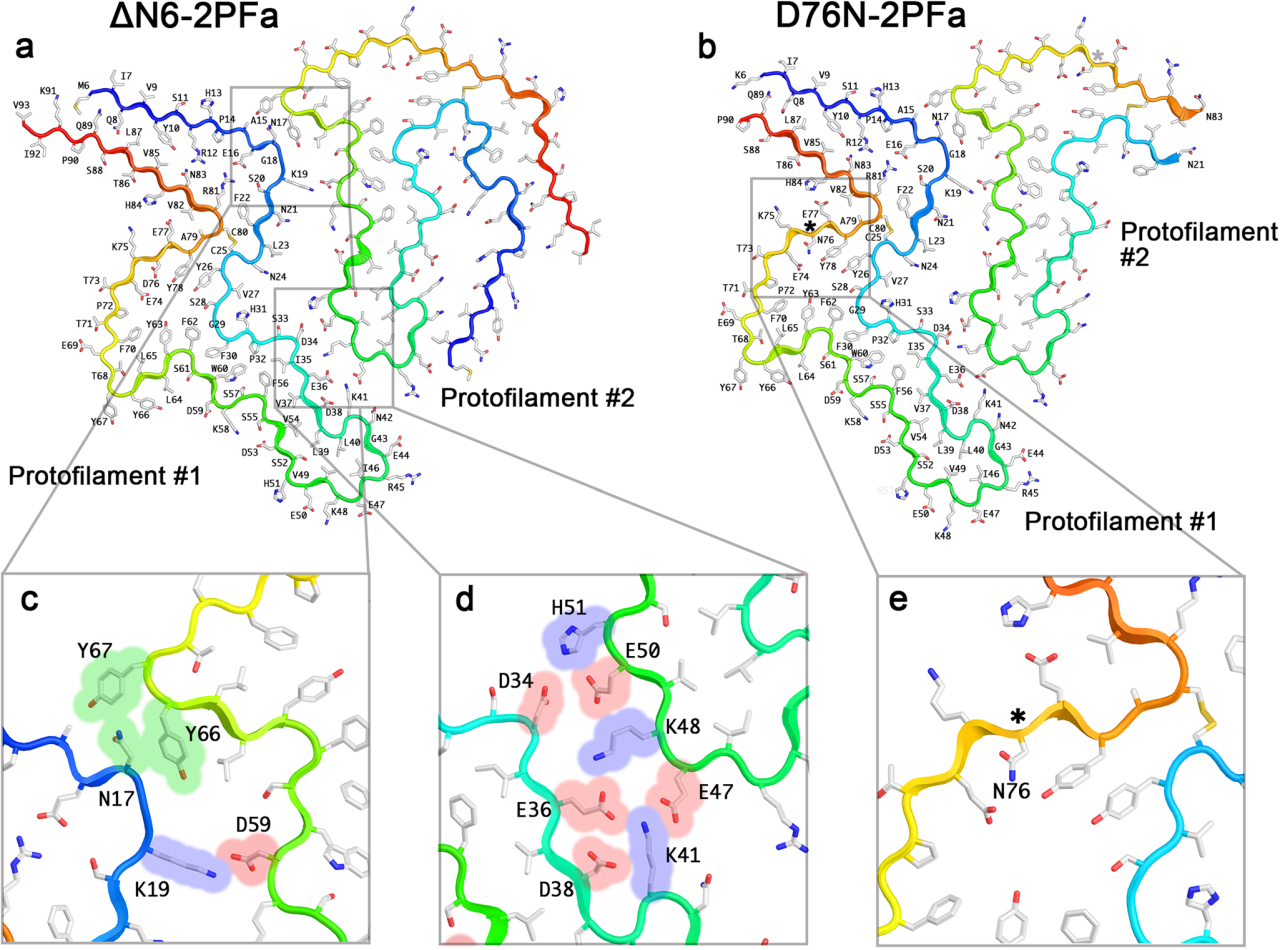
Side-by-side polymorphs are built using polar/charged interactions and require an ordered N- and C-terminus. The atomic models for **a**, β_2_m-ΔN6-2PFa and **b**, β_2_m-D76N-2PFa are shown in cartoon representation, with the backbone cartoon coloured from N- to C- terminus (blue to red). For each, sidechains are shown in stick representation and coloured as CPK. Residues in protofilament #1 are explicitly labelled. Interactions that hold the two filaments together are similar in each structure, including a patch of polar and charged residues (**c**), and a series of salt-bridge interactions (**d**). The Aspartate→Asparagine substitution is within the amyloid core, and accommodated without discernible perturbation of the structure of the fold (**e**). For **c**–**e**, the space-filling footprint of the amino acid side chain is shown as a transparent colour: green=polar, red=acidic, blue=basic.

The ΔN6-2PFa and D76N-2PFa structures have a very similar structure (Fig. 7a, b). In each, protofilament #1 adopts a “termini up” conformation, whilst protofilament #2 adopts a “termini down” orientation. There are two interaction surfaces between the protofilaments. Firstly, the”termini-up” conformation of protofilament #1 presents a peptide segment (residues 15–19) that allows the formation of a binding interface with protofilament #2. This involves both polar (residue N17 interacts with Y66 and Y67 in the adjacent protofilament (Fig. 7c, Supplementary Fig. 9)), and charge-based interactions (including a salt-bridge between K19 and D59 (Fig. 7c, Supplementary Fig. 9)). Secondly, a series of electrostatic interactions occurs between residues 34–41 in protofilament #1 and residues 47–51 in protofilament #2, which stabilise the fibril structure (Fig. 7d, Supplementary Fig. 9).

Interestingly, the N- and C-termini are less ordered in the β_2_m-D76N fibril structures compared with those observed in the β_2_m-ΔΝ6 fibrils. In protofilament #1, this is only a minor effect, with residues 6–93 being resolved for β_2_m-ΔΝ6 fibrils (the natural N-terminus), while residues 6–90 are visible in fibrils of β_2_m-D76N. The difference in termini is more notable in protofilament #2, in which residues 6–93 are again visible in the β_2_m-ΔN6 fibril structure, but only residues 21–83 are resolved (i.e. only ~62% of the primary sequence) in the β_2_m-D76N fibril. Notably, this marked difference in dynamics of the termini of β_2_m-D76N fibrils occurs despite the absence of any obvious perturbation of the amyloid core structure caused by the substitution of Asp76 for Asn, since residue 76 is located within a polar cavity that accommodates either side chain without generating steric hindrances or electrostatic repulsion (Fig. 7e).

### Tail-to-tail polymorphs assemble without the involvement of the N- and C-termini

**Fig. 8:**
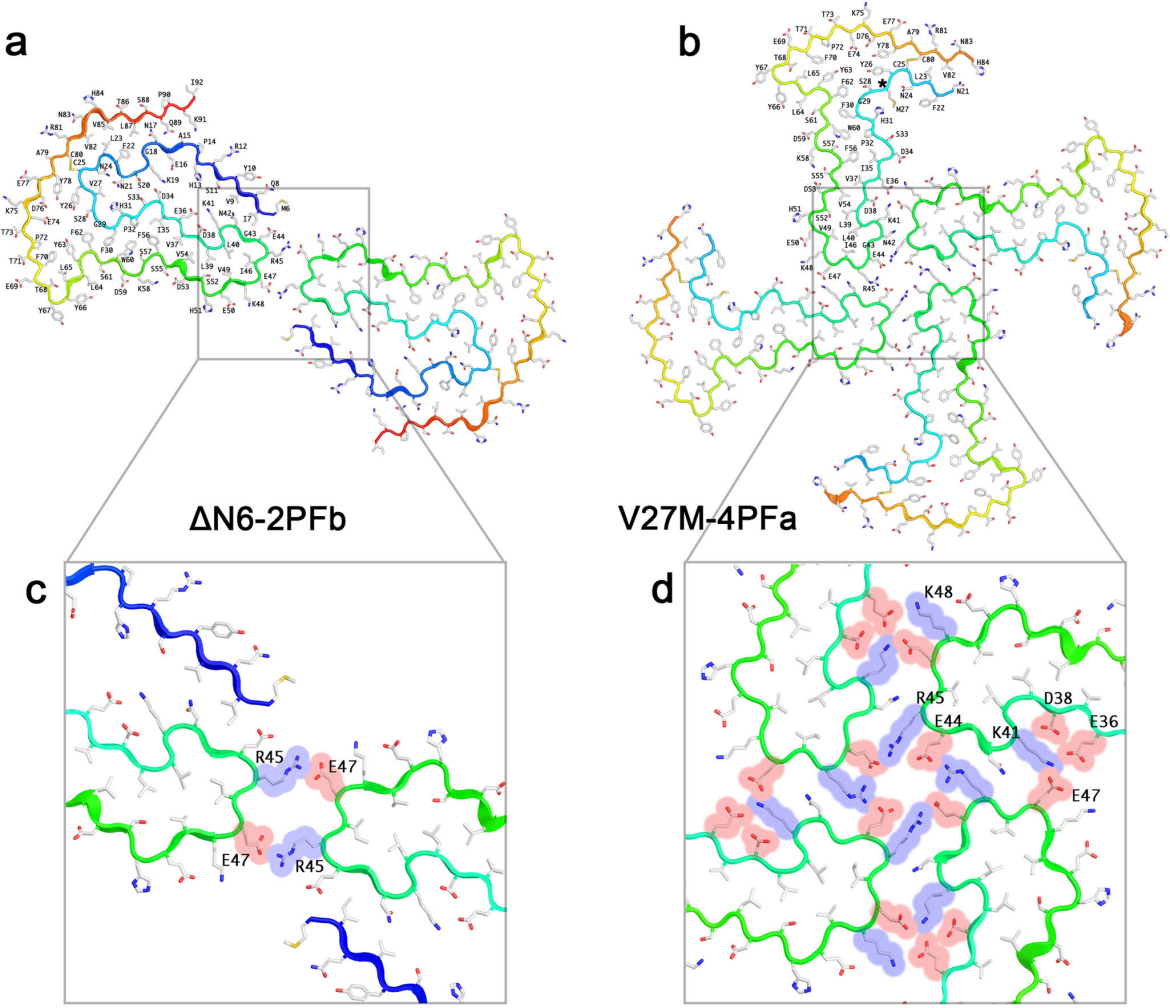
Base-to-base fibril polymorphs are built using salt-bridge interactions. The atomic models for **a**, ΔN6-2PFb and **b**, V27M-4PFa are shown in cartoon representation, with the backbone cartoon coloured from N- to C- terminus (blue to red). For each, sidechains are shown in stick representation and coloured as CPK. Residues in protofilament #1 are explicitly labelled. For the ΔN6−2PFb structure **c**, the protofilaments are held together by a pair of salt-bridge interactions (R45/E47), whilst for the V27M-4PFa structure **d**, E44/R45 form the salt bridges. In V27M-4PFa, additional salt-bridge interactions stabilise the fibril. For **c**, **d**, the space-filling footprint of the amino acid side chains involved in the intersubunit interfaces are shown as a transparent colour, with acidic residues in red and basic residues in blue.

The ΔN6-2PFb and V27M-4PFa structures have different quaternary structures, but are built using similar salt-bridge interactions between charged residues at the tail of each subunit (Fig. 8a, b, Supplementary Figs. 9, 10). In ΔΝ6-2PFb, each of the two identical filaments contributes two charged residues, R45 and E47, which form a pair of intermolecular salt bridges that stabilises the two protofilament conformation (Fig. 8c). This polymorph represents a minority of the fibrils in the β_2_m-ΔΝ6 fibril dataset, and the area buried in its intersubunit interface is smaller (~80 Å^2^ / layer) compared to ΔΝ6-2PFa (179 Å^2^ / layer), suggesting that the ‘tail-to-tail’ arrangement may be less stable than its ‘side-by-side’ counterpart. Indeed, thermodynamic stability calculations, as employed by the Amyloid Atlas^12^, estimates a slightly lower ∆G^o^ for forming a fibril layer of ∆N6-2PFb compared to ∆N6-2PFa, although all of the β_2_m fibril structures from this study have near identical stabilities when calculating the average per residue contribution (Supplementary Fig. 11). This suggests that the majority of the fibril stability comes from intra- rather than inter-protofilament interactions, and may explain why the same stabilising core fold can be arranged in different quaternary arrangements that are presumably promoted by subtle changes induced by the mutations studied here. The subunits in both protofilaments of ΔΝ6-2PFb are in a ‘termini down’ conformation, but the termini take no part in the intersubunit interface.

For V27M-4PFa, the ‘tail-to-tail’ arrangement is similar to that seen for ΔΝ6-2PFa, with residue R45 involved in each, alongside E44, forming a pattern of alternating basic and acid interactions at the heart of the fibril assembly. Additionally, a second contact patch that also involves charged residues is made between each protofilament (Fig. 8d), with the interaction between protofilament #1 and #2 being more extensive than that between protofilaments #2 and #3 (resulting in the C2 symmetry of this polymorph). Each protofilament has disordered N- and C-termini, with only residues N21-H84 being sufficiently ordered to build an atomic model. However, it is clear from an examination of the low-resolution density at the periphery of the map, that the termini are in an ‘up’ conformation, and indeed there would not be enough room to accommodate the termini in a ‘down’ conformation in the densely-packed 4PF structure.

### The fold of the β_2_m variants at near-neutral pH is different to that of the WT-β_2_m fibrils at acidic pH

The hammer motif that is observed in all of the β_2_m variant fibril structures shown here is different to that observed previously for WT-β_2_m fibrils^32^ formed at pH 2.5, despite a superficially similar subunit shape (Supplementary Fig. 12). The chain of both folds threads a similar path from the disulphide bond that anchors each structure, however subtle kinks in the chains result in dramatic changes in the registers between amino acid side chains in the amyloid core that are very different between the folds (Supplementary Fig. 12a). This is highlighted when comparing the internal distances between matching residue pairs in the structures (e.g.: Cα distance between the residue pairs F30-W60 and L40-E50 respectively: 10 Å and 12.5 Å in the neutral pH fold vs 32 Å and 24 Å in the WT-β_2_m acidic pH fibril fold). The cumulative differences result in a substantial overall RMSD of 11.2 Å between Cα’s for residues 25–80 in the near-neutral and acidic pH fibrils, and a number of residues flip between side chains pointing into the amyloid core or pointing towards solvent, and vice versa (Supplementary Fig. 12b). This is particularly evident in the region of the ‘head’ of the hammer motif, where the acid and near neutral β_2_m amyloid folds are most different. Small segments are similar between the folds, but their superposition confirms the extent of the conformational rearrangements in the rest of the core (Supplementary Fig. 12c). Finally, the inter-protofilament interface of WT-β_2_m fibrils at acidic pH, involving Y67 in one chain and E69 in the other, is not seen in the neutral pH β_2_m variant fibril structures. We can speculate why the acidic pH WT-β_2_m fibril structure is disfavoured at neutral pH, as in the WT, acidic pH fold, E44 and D76 are buried in internal hydrophobic cavities which is predicted to be highly destabilising in ∆G^o^ thermodynamic stability calculations^12^ at neutral pH where the acidic moieties would be charged (Supplementary Fig. 11). The fold of the β_2_m variants at pH 6.2 appears to have adjusted to alleviate this destabilisation: E44 flips out of the intramolecular core to be solvent exposed and a less-well packed hammer-head is created to accommodate the polar and charged residues in an internal, solvent filled cavity (Supplementary Fig. 7). Interestingly, the ∆G^o^ analysis suggests that E74 and D76 are still destabilising in this conformation, although this does not include potential stabilisation by water molecules that are not resolved in the structure. This analysis does however predict that the D76N substitution would completely negate the destabilising effect of this residue in the amyloid fold, which may explain why D76N fibrils form faster than the other variants (Fig. 1).

## Discussion

A growing body of literature now describes the structures of amyloid fibrils that display a breath-taking diversity of ways in which the canonical cross-β amyloid fold can be realised in three-dimensions^12^. However, if a consensus is emerging, it is that ex vivo fibrils are more homogeneous than those formed in vitro^13,16,17,48,55^. This knowledge has arisen with the striking observation that different proteinopathies caused by deposition of the same protein/peptide sequence are characterised by different amyloid folds. This has been shown compellingly for both Αβ and tau fibrils extracted ex vivo from brain tissue donated by people with a range of neurodegenerative disorders^13–16^. Equally striking are in vitro studies which have shown that small variations in the assembly conditions enable tau to achieve an array of amyloid folds, some of which are identical to those observed in vivo^7,56^.

The β_2_m variants studied here in vitro have different effects in vivo, with distinct human pathology associated with each: an iatrogenic joint arthropathy for β_2_m-ΔN6; a familial, systemic amyloidosis for β_2_m-D76N; and an iatrogenic deposition in the tongue and salivary glands for β_2_m-V27M. It is tempting, therefore, to hypothesise that the self-assembly of these different variants would result in different amyloid folds, especially given recent data showing that the ‘arctic’ sequence variant of Aβ (E22G) forms a different fibril structure ex vivo^57,58^ than that observed for the WT-Aβ sequence^16^. The data presented here suggests this may not necessarily be the case for β_2_m and its variants. The fibrillation reactions we describe produce fibrils that are polymorphic, but we show that this polymorphism arises from the intermolecular association of a strikingly conserved building block. Determining whether this is the case in vivo will require purification of amyloid ex vivo from the different β_2_m amyloid disorders, although their rarity renders this a significant challenge. It could also be that currently uncharacterized changes in tissue microenvironment, such as changes in ionic homeostasis prompted by long term dialysis (for β_2_m-ΔΝ6 and β_2_m-V27M), or the specific local microenvironment of the viscera (β_2_m-D76N) may also play a role in determining the fibril structure formed in a physiological setting. We also note the very recent discovery of another β_2_m variant (P32L^59^) that leads to a different systemic amyloidosis, and it would be fascinating to determine whether the same core fold and fibril polymorphism is observed in that case also, especially given the known importance of cis-trans isomerisation of the proline at this position in β_2_m folding and aggregation^53^.

More broadly, understanding proteinopathies, and potentially intervening in their progression therapeutically, will require a vastly improved understanding of the richness of amyloid polymorphism in tissue and how it changes with changes in protein sequence, and cellular environment, and time. Given the rareness of the disorders associated with β_2_m amyloid deposition, and the consequent difficulty in obtaining biological samples, this remains a prodigious undertaking, and will likely critically depend on good model systems that faithfully reproduce in vitro the aggregation that occurs in diseased tissue.

## Methods

### Cloning, gene expression and protein production

The β_2_m-ΔN6, β_2_m-D76N and β_2_m-V27M genes were independently cloned into the pINK plasmid using the NdeI and HindIII restrictions sites respectively. For the β_2_m-ΔN6 gene, the first six residues of wt-β_2_m were removed and an N-terminal methionine was introduced prior to Ile7 to facilitate gene expression^37^. Gene expression and protein production was carried out using *E. coli* BL21(DE3) cells, with induction using 1 mM of IPTG at an OD_600_ of 0.7, followed by 12 h of growth at 37 °C. Cells were harvested by centrifugation and the pellets were processed immediately. The three variants were separately purified as described previously^36^. Finally, the protein samples were aliquoted and snap frozen in liquid nitrogen for storage at −80 °C.

### Fibrillation reactions

To generate fibrils of WT- β_2_m, β_2_m-ΔN6, β_2_m-D76N and β_2_m-V27M for ThT and EM studies, an aliquot of each frozen protein was thawed and passed through a Superdex 75 26/600 (GE healthcare) size exclusion chromatography column, previously equilibrated with fibrillation buffer (25 mM sodium phosphate, 115 mM NaCl, pH 6.2). Each eluted monomeric peak was collected and adjusted to the final β_2_m monomer concentration desired and used immediately. For ThT fibril growth assay, five replicate reactions were set for each protein in Costar 96-well plates (Corning) using a Fluostar Omega (BMG Labtech) plate-reader. Reaction volumes were 100 µL, with 40 µM of each protein and 10 µM ThT in the fibrillation buffer. The ThT reactions were run for 100 h at 37 °C with 600 rpm shaking for 5 min at 6 min intervals and the fluorescence for each individual run was normalised to an arbitrary scale of 0 to 1 for plotting. For structure determination, 20 µM (β_2_m-ΔN6 and β_2_m-V27M) or 40 µM (β_2_m-D76N) of protein in 1 mL fibrillation reactions in 1.5 mL Eppendorf tubes were incubated at 37 °C with continuous shaking at 600 rpm in a benchtop Eppendorf thermomixer. Fibrils were visible by negative stain EM after 72 h.

### Negative stain EM

A 50 µL aliquot of each fibrillation reaction was pelleted by centrifugation (10 min at 10,000 × *g*). The pellet was resuspended in 500 µL of MilliQ water and the centrifugation step repeated. The pellet was then subjected to a brief wash with acidified water by resuspension in 500 µL of 6 mM HCl to disentangle the fibrils and reveal crossovers amenable to structure determination by helical averaging (Supplementary Fig. 13). Centrifugation was repeated and the pellet resuspended in 50 µL of the resulting supernatant. Grids were prepared immediately so that the total exposure time of the fibrils to the acidic pH was <15 min, a timescale on which new fibril formation is unlikely occur. Based on ThT data in acidic conditions, no new fibrils form in this time period. 4 µL of each acid-washed reaction was added to 300 mesh carbon grids, and left for ~1 min prior to blotting. The grid was then washed twice with distilled water, and then stained with 1% (w/v) uranyl acetate. Grids were imaged using a Technai F20 EM with a Ceta CMOS detector (Thermo Fisher Scientific) at magnifications of 1000–50,000×.

### CryoEM sample preparation

The β_2_m-ΔN6, β_2_m-D76N and β_2_m-V27M fibrillations were briefly acid washed, as described above, immediately prior to grid making. For β_2_m-ΔN6, plasma-cleaned 300 mesh lacey carbon grids were coated with two applications of a 0.02 mg/mL graphene oxide, 0.3 mM n-Dodecyl-Beta-Maltoside (DDM) solution as described in other studies^60,61^. For β_2_m-V27M, graphene oxide was similarly used with plasma-cleaned 300 mesh Quantifoil R 1.2/1.3 grids. In each case, a sample volume of 4 µL was applied to the graphene oxide and coated carbon-side of the grid. For β_2_m-D76N, a sample volume of 3 µl aliquot was applied directly to a plasma-cleaned lacey carbon 300 mesh copper grid. All of the samples were frozen by plunging in liquid ethane using a Vitrobot Mark IV (FEI) with a 4–6 s blot time. The Vitrobot chamber was maintained at close to 100% humidity and 4 °C.

### CryoEM data collection

CryoEM datasets for all three variants were collected using a Titan Krios microscope operated at 300 kV, with a Falcon4 detector in counting mode and EPU 3.0 data collection software (Thermo Fisher Scientific), at a nominal magnification of 96,000× (0.83 Å/pixel) for β_2_m-ΔN6 and β_2_m-D76N. A Selectris energy filter operating at 10 e^−^V, and paired with a Falcon4 detector, was additionally used for collection of the β_2_m-V27M dataset with a nominal magnification of 130,000× corresponding to 0.94 Å/pixel. For β_2_m-ΔN6, a total of 4095 images were collected with a nominal defocus range of −1.3 to −2.5 µm in 0.2 µm increments. Each image consisted of an EER movie stack with an accumulate dose of ~43 e^−^/Å^2^ across a 7 s exposure, corresponding to a dose rate of ~4.4 e^−^/pixel/s. For β_2_m-D76N, a total of 3,849 images were collected with a nominal defocus range of −0.8 to –3.0 µm in 0.2 µm increments. Each EER movie stack was collected with a total dose of ~43 e^−^/Å^2^, exposure of 6.2 s, and a dose rate of ~5.2 e^−^/pixel/s. For β_2_m-V27M, a total of 4,079 images were collected with a nominal defocus range of −1.3 to –2.5 µm in 0.3 µm increments. Each EER movie stack was collected with a total dose of ~43 e^−^/Å^2^, exposure of 6 s, and a dose rate of ~5.9 e^−^/pixel/s.

### β_2_m-ΔN6 cryoEM data processing

For β_2_m-ΔN6, each movie stack was grouped from 1687 raw EER frames into 40 fractions (~1.1 e^−^/Å^2^ per fraction) and aligned and summed using motion correction in RELION4^62^. CTF parameters were estimated for each micrograph using CTFFIND v4.14^63^. Poor quality images, based on CTF figure of merit, defocus value and predicted resolution were removed, yielding 3880 micrographs for further processing. Fibrils from 112 micrographs were manually picked in RELION and the extracted segments used to train automated filament picking in crYOLO^64^. Using an inter-box spacing of 15 Å, a total of 737,827 helical segments were extracted (2x binned) in RELION4 with box dimensions of 540 Å. 2D classification with the EM algorithm in RELION4 was used to remove picking artefacts and unfeatured objects, leaving 619,671 fibril segments. Three fibril polymorphs were evident within the 2D class averages, so the data was split into three pools (Supplementary Fig. 1a). This resulted in 423,385 segments (69% of those selected) selected for 2PFa, the major species, which showed two asymmetrically-arranged protofilament fibrils, 113,783 segments (18% of those selected) for 1PF which showed single-protofilament fibrils and 82,503 segments (13% of those selected) for 2PFb which showed symmetric two-protofilament fibrils. Each separate pool was further cleaned by 2D classifying again, then a final time after unbinned extraction with 270 Å box dimensions.

The cleaned 413,422 2PFa segments were used for 3D classification using a 15 Å low-pass filtered initial template generated from the processing of a similar form within the β_2_m-D76N dataset, with a corresponding helical twist and rise of 359.01˚ and 4.85 Å respectively (Supplementary Fig. 1b). From this, two classes showing a conserved traceable peptide backbone were selected containing a total of 288,510 segments. Multiple sequential 3D refinements were needed to improve the helical parameter estimates until a map was obtained at a resolution of 3.5 Å (all resolutions are RELION gold-standard with 0.143 FSC cut-off) with a helical twist of 359.15˚ & rise of 4.85 Å. Per-particle CTF refinement and Bayesian polishing improved the resolution to 3.1 Å (Supplementary Fig. 1c). The core repeating unit of this form represented two protomers with a similar core fold, but with variations in the position of their N- and C-termini. The density for the termini of one of the protomers in the asymmetric unit was weaker, but further classification without angular searches separated a class with ordered termini for the second protomer (Supplementary Fig. 1d). These 132,469 segments were refined to give the final β_2_m-ΔN6 2PFa map at a resolution of 3.0 Å with a sharpening B-factor of −72 Å^−2^ applied prior to deposition and a refined helical twist of 359.18˚ and rise of 4.85 Å (Supplementary Fig. 1e).

For the β_2_m-ΔN6 1PF segments, an initial crossover length could not be estimated from either the raw micrographs or 2D class averages. The cleaned 78,209 Form1 segments were 3D classified with multiple templates and helical parameter searches, but the structure could not be solved to high resolution. The fibril core that emerged from the classifications resembles the same overall fold of the 2PFa fibrils but with just a single protomer per layer instead of two (Supplementary Fig. 1f). The cleaned 48,531 β_2_m-ΔN6 2PFb segments were 3D classified with multiple templates and helical parameter searches until a solution with a defined peptide backbone began to emerge. With a helical twist of 359.20˚ and rise of 4.85 Å, the resulting 3D classification separated 43,706 segments into two classes in which the backbone was visible (Supplementary Fig. 1g). After multiple subsequent refinements. the optimised helical rise and twist were determined to be 359.41˚ and 4.86 Å respectively. A second 3D classification was then run where a single class with 11,324 segments and separation of ß-strands was selected (Supplementary Fig. 1h). These segments refined to a resolution of 3.8 Å. After per-particle CTF refinement, Bayesian polishing and a final 3D classification without image alignment, 10,594 segments classifying into the highest resolution class. These refined to give a map at a resolution of 3.4 Å (Supplementary Fig. 1i). The repeating core of this form contained two superficially identical protomers with a fold that was almost indistinguishable from one of the protomers in the 2PFa structure. Refining with C2 symmetry applied however led to breaks in the backbone density, so the final 2PFb map was deposited with C1 symmetry and a sharpening B-factor of −37 Å^2^. The local resolution of both the 2PFa and 2PFb maps (and indeed all of the high-resolution structures determined herein) was calculated and is show in Supplementary Fig. 14.

### β_2_m-D76N cryoEM data processing

For β_2_m-D76N, each of the movie stacks was grouped from 1477 frames into 54 fractions (~0.8 e^−^/Å^2^ per fraction) and aligned and summed using motion correction in RELION3.1.1^65^. CTF parameters were estimated for each micrograph using CTFFIND v4.14. Fibrils from 61 micrographs were manually picked in RELION and the extracted segments used to train automated filament picking in crYOLO. Using an inter-box spacing of 5 Å, a total of 1,117,062 helical segments were extracted 3x binned in RELION3.1 with box dimensions of 660 Å. This large particle dataset was split in 4 subsets, each containing 279,264 particles. Each subset was separately subjected to 2D classification to remove picking artefacts and unfeatured objects, leaving a total of 974,932 fibril segments. A second round of 2D classification facilitated the separation of segments into two main categories according to the apparent fibril width, resulting in a combined total of 574,659 thin fibril segments (1PF) and 400,273 wide fibril segments (2PF) (Supplementary Fig. 3a). A subclass of wide-fibril segments displaying a helical cross-over of 96 nm was used to generate an initial model using relion_helix_inimodel2d^66^. This model was employed as a template for 3D classification, which allowed separation of the wide fibrils into 2 classes (Supplementary Fig. 3g). The particles corresponding to each of these classes were individually selected and re-extracted unbinned with a box size of 220 Å for further 3D classification rounds, using the corresponding 3D-class model as a starting template. One of these classes (2PFb) was not solvable (Supplementary Fig. 3h). The second class was equivalent to the β_2_m-ΔN6 2PFa fibril structure and, after multiple refinements with helical parameter searches, the 78,097 segments refined to a resolution of 3.6 Å. After CTF refinement, Bayesian polishing and 3D refinement, the final β_2_m-D76N 2PFa map was solved at a resolution of 3.0 Å and deposited with a sharpening B-factor of −30 Å^2^ (Supplementary Fig. 3i). The refined helical parameters were a twist of 359.01˚ and rise of 4.80 Å respectively.

A similar strategy was followed for the initial 3D-classification of the β_2_m-ΔN6 thin fibril segments, from which two different classes were obtained and labelled as 1PFa and 1PFb respectively (Supplementary Fig. 3b). The particles corresponding to each of these classes were selected individually and re-extracted unbinned in 220 Å boxes. For D76N-1PFa, two subsequent rounds of 3D classification with searches of the helical parameters led to selection of 44,011 ordered segments with a helical twist of 358.57˚ and rise of 4.80 Å (Supplementary Fig. 3c). For D76N-1PFa, a single additional round of 3D classification with helical searches led to the selection of 72,815 ordered segments with a helical twist of 358.49˚ and rise of 4.80 Å (Supplementary Fig. 3e). However, even after CTF refinement and Bayesian polishing of both subsets, the refined 1PF maps continually showed regions of the backbone density where the layers were artificially fused (Supplementary Fig. 3d, f) and so the maps could not be deposited as resolved fibril structures. The fold of the repeating fibril cores for 1PFa and 1PFb clearly showed single protofilament structures resembling the core fold of the other β_2_m structures in this study, but with varying distinct positions of the N- and C-termini.

### β_2_m-V27M cryoEM data processing

For β_2_m-V27M, each of the movie stacks was grouped from 1442 frames into 40 fractions (~1.1 e^−^/Å^2^ per fraction) and aligned and summed using motion correction in RELION4. The fibril density was lower than the other variants, therefore, lowpass filtered micrographs were screened for the presence of fibrils resulting in the selection of 611 micrographs for further processing. CTF parameters were estimated for each micrograph using CTFFIND v4.14. Fibrils from 83 micrographs were manually picked in RELION and the extracted segments used to train automated filament picking in crYOLO. Using an inter-box spacing of 15 Å, a total of 229,692 helical segments were extracted 2× binned in RELION with box dimensions of 564 Å. Two rounds of binned 2D classification using RELION4’s VDAM gradient algorithm were used to remove picking artefacts and unfeatured objects to leave 138,540 segments for unbinned extraction with 280 Å box dimensions. After another two rounds of 2D classification with the EM algorithm, 112,947 fibril segments resembling amyloid fibrils were selected and split into two subsets: 43,554 thin fibril segments and 69,393 wide fibril segments (Supplementary Fig. 4a).

The wide fibril segments appeared different to any of the forms seen for the other two variants and so were 3D classified using an initial model generated from a single 2D class average with the relion_helix_inimodel2d command and an estimate of the crossover from binned 2D class averages. The resulting classes showed a potential 4PF propellor-like structure with the most ordered class selected containing 33,566 segments (48% of those classified, Supplementary Fig. 4b). After additional 3D classification with searches of the helical twist, a homogeneous class containing 4,732 segments was selected (Supplementary Fig. 4c) which contained improved density for discerning the peptide backbone in the fibril core. Up until this point no additional symmetry had been applied, so 3D refinements were attempted with C1, C2 and C4 symmetry (Supplementary Fig. 4d). The best map with continuous density for the peptide backbone consistently came from the C2-symmetrised maps, with the backbone density becoming broken on the outer regions of the fibril core when C4 symmetry was applied. As a result, the 4732 segments were CTF refined and Bayesian polished, followed by 3D refinement to give the final V27M-4PFa map for deposition at a resolution of 2.8 Å with a sharpening B-factor of −38 Å^2^ (Supplementary Fig. 4e). The refined helical parameters were a twist of 359.27˚ and rise of 4.85 Å respectively.

Multiple 2D classification attempts were run with the 43,554 thin fibril segment pool, however the lack of features obtained in 2D class averages frustrated polymorph identification attempts (Supplementary Fig. 4g). A similar outcome was seen from 3D classification attempts using the solved ΔN6 and D76N fibril structures and associated helical parameters (Supplementary Fig. 4h). As such, composition and structural information could not be determined for the thinner fibril population(s) accounting for 39% of the total cleaned fibril segments in the data. The different fibril widths observed in 2D class averages are however consistent with 1PF and 2PF fibrils when comparing to the ΔN6 and D76N datasets respectively.

### Model building and refinement

Despite having a similar superficial appearance, the published wt-β_2_m structure of fibrils grown at pH 2 (EMDB-0014; PDB-6gk3^32^) did not fit well into the maps of either β_2_m-ΔN6 and β_2_m-D76N from neutral pH fibrillations. Therefore, the highest resolution map, ΔN6-2PFa at 3.0 Å, was used to de novo build the peptide model for one layer of the fibril core using Coot^67^. One protomer was built first and then used to guide building of the second protomer as their folds were very similar between residues Cys25-Cys80. Both Ramachandran and rotamer outliers were monitored and minimised during building in Coot. The final built layer was then repeated and rigid body fit to generate a model for 3 layers of the fibril core, which was then used for real space refinement against the deposited map using Phenix v1.17.1^68^. NCS restraints were applied to prevent divergence of repeating chains in the layers.

A similar approach was then used for modelling the ΔN6-2PFb, D76N-2PFa and V27M-4PFa structures, but in each case starting with the ΔN6-2PFa structure as an initial template. The final real space refined models for each structure were assessed using MolProbity^69^. The final model statistics for all structures solved at high resolution are summarised in Table 1.

**Table 1:** Cryo-EM data collection, refinement and validation statistics for the β_2_m DN6 dataset

	β_2_m-∆N6 2PF_A_ (EMDB-15222) (PDB 8a7o)	β_2_m-∆N6 2PF_B_ (EMDB-15223) (PDB 8a7p)	β_2_m-D76N 2PF_A_ (EMDB-15225) (PDB 8a7t)]	β_2_m-V27M 4PF (EMDB-15224) (PDB 8a7q)
**Data collection and processing**				
Magnification	96,000		96,000	130,000
Voltage (kV)	300		300	300
Detector	Falcon4		Falcon4	Falcon4-Selectris
Pixel size (Å)	0.83		0.83	0.94
Electron exposure (e^–^/Å^2^)	43		43	43
Exposure rate (e^–^/pixel/s)	4.4		5.2	5.9
Nominal defocus range (μm)	−1.3 to −2.5		−0.8 to −3.0	−1.3 to −2.5
Movies collected	4095		3849	4079
Initial particle images (no.)	612,949		1,117,062	229,692
Final particle images (no.)	133,576	10,594	78,097	4732
Symmetry imposed	C1	C1	C1	C2
Map resolution (Å) FSC threshold	3.0 0.143	3.4 0.143	3.0 0.143	2.8 0.143
Map resolution range (Å)	2.9–5.5	3.3–6.8	3.0–6.6	2.8–4.8
Helical parameters				
Helical twist (°)	359.18	359.40	359.01	359.27
Helical rise (Å)	4.85	4.86	4.80	4.85
Crossover (nm)	106	145	87	120
**Refinement**				
Initial model used (PDB code)	–	8a7o	8a7o	8a7o
Map sharpening *B* factor (Å^2^)	−72	−37	−30	−38
Model to map correlation	0.88	0.78	0.85	0.79
Model composition				
Non-hydrogen atoms	4356	4314	3696	6480
Protein residues total	528	522	444	768
Protein residues modelled	6–93(A,B)	6–92(A,B)	6–90(A), 21–83(B)	21–84(A,B,C,D)
Chains per helical layer	2	2	2	4
Helical layers modelled	3	3	3	3
*B* factors (Å^2^)				
Protein	53	81	86	41
R.m.s. deviations				
Bond lengths (Å)	0.002	0.003	0.003	0.004
Bond angles (°)	0.540	0.535	0.454	0.667
Validation				
MolProbity score	1.5	1.9	1.4	1.6
Clashscore	6.7	6.4	5.0	7.8
Poor rotamers (%)	0.0	1.9	0.0	0.0
Ramachandran plot				
Favored (%)	97.1	95.3	97.2	96.8
Allowed (%)	2.9	4.7	2.8	3.2
Disallowed (%)	0.0	0.0	0.0	0.0

### AFM imaging

For β_2_m-ΔN6, β_2_m-D76N and β_2_m-V27M, fibrils were diluted to 10 μM in water and 1 μl pH 1 HCl was added to 19 μl of fibril sample. The sample was incubated on freshly cleaved mica for 15 min then washed with 1 ml MilliQ water and dried with a stream of nitrogen. The sample was imaged on a multimode 8 (Bruker) with a Bruker Scanacyst air probe. Height channel images were collected with one of the following sets of parameters, 2 × 2 μm squares with 1024 × 1024 pixels and a scan rate of 0.488 Hz, 4 × 4 μm with 2048 × 2048 pixels and a scan rate of 0.203 Hz or 8 × 8 μm with 4096 ×4096 pixels and a scan rate of 0.203 Hz. A noise threshold of 0.5 nm was used throughout. The peak force set point was set to not exceed 400 pN. This was achieved by calculating the spring constants for 10 Scanacyst air probes by acquiring force distance curves on clean mica discs and finding the corresponding voltage required to achieve 400 pN of force in the stiffest cantilevers in that range. The voltage set point used was then set below this value. Nanoscope analysis software (Version 1.5, Bruker) was used to process the image data by flattening the height topology data to remove tilt and scanner bow. Fibrils were traced and digitally straightened and height profiles were extracted across the centre line of each fibril in Matlab (R2020a). Fibrils were traced and digitally straightened and height profiles were extracted across the centre line of each fibril in Matlab as described in^70^. 3D models from the AFM data were generated following the methodology in Lutter et al.^71^.

### Reporting summary

Further information on research design is available in the Nature Portfolio Reporting Summary linked to this article.

## Supplementary information

Supplementary Information

Reporting Summary

## Data Availability

The three raw cryoEM datasets are available in the EMPIAR database as: β_2_m-ΔΝ6 (EMPIAR-11381), β_2_m-D76N (EMPIAR-11384) and β_2_m-V27M (EMPIAR-11383). Refined EM maps and atomic models are deposited in the EMDB and PDB respectively (where an atomic model was built) as: ΔΝ6-2PFa (EMDB-15222/PDB-8a7o), ΔΝ6-2PFb (EMDB-15223/PDB-8a7p), D76N-1PFa (EMDB-15226), D76N-1PFb (EMDB-15227), D76N-2PFa (EMDB-15225/PDB-8a7t) and V72M-4PFa (EMDB-15224/PDB-8a7q). Raw data from this study are available at the University of Leeds Data Repository: 10.5518/1292. Source data are provided with this paper.

## Source data

Source data
