# Supplementary material for: Disease-relevant β2-microglobulin variants share a common amyloid fold
Source: Nat Commun. 2023 Mar 2;14:1190. doi: 10.1038/s41467-023-36791-8 (PMC9981686; doi:10.1038/s41467-023-36791-8)
Supplement: Supplementary file 1 — Supplementary Information [file 41467_2023_36791_MOESM1_ESM.pdf]

Disease-relevant β_2-microglobulin variants share a common amyloid fold - Supplementary Data

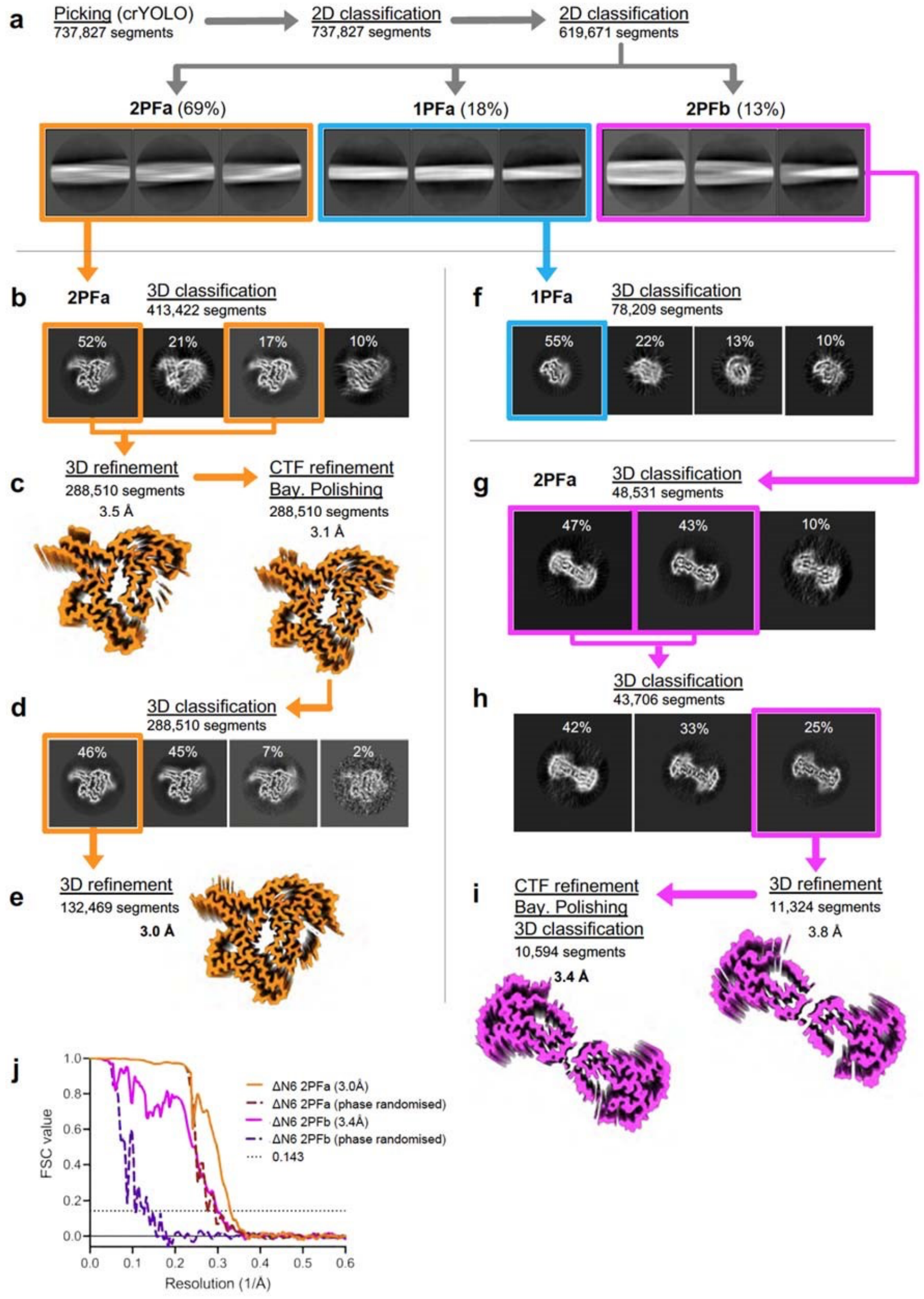

Supplementary Figure 1. Processing summary for the β_2m-ΔN6 cryo-EM dataset.
a, Summary of the initial processing steps for the β_2m-ΔN6 cryo-EM dataset. After multiple rounds of cleaning by 2D classification, the dataset was split into three fibril polymorphs with example 2D class averages from 2x binned segments displayed for each form. **b-d**, The major form 2PFa was further processed as shown, for all 3D classification runs shown, the resulting output classes are shown as sums of the central 6x slices through the fibril map to visualise roughly one amyloid layer (i.e. the 4.8Å repeating slab of density). **e**, The final 2PFa map was solved to 3.0 Å resolution, displayed with a sharpening value of -72 Å^2. **f**, The 1PF fibril segments could not be resolved to high resolution but 3D classification showed a related peptide fold to those present in the 2PFa form. **g-h**, The 2PFb fibril segments were further processed as shown. **i**, The final 2PFb map was solved to 3.3 Å resolution, displayed with a sharpening value of -37 Å^2. **j**, FSC plots of the two solved β_2m-ΔN6 fibril structures with corresponding phase randomised FSC curves as dashed lines. Source data are provided as a Source Data file.

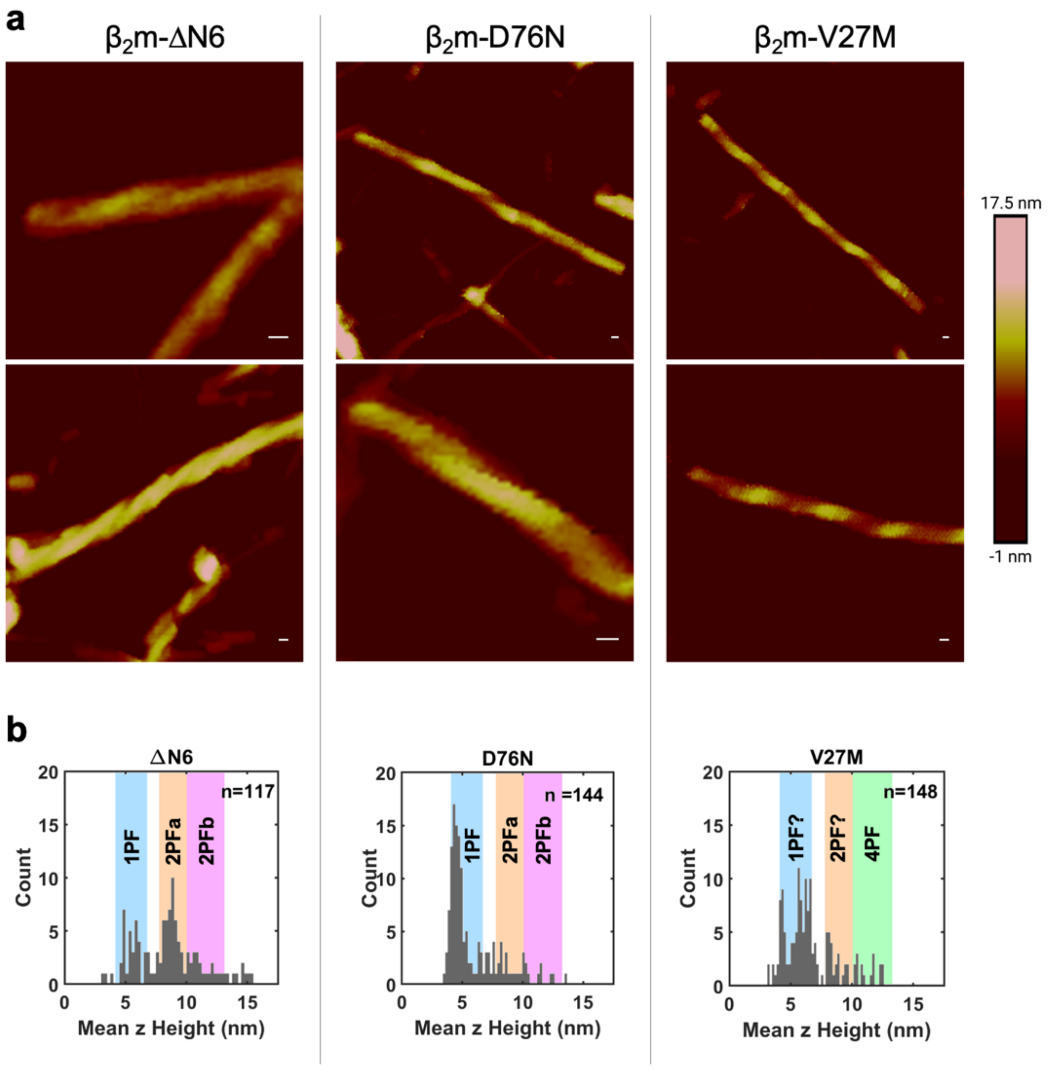

Supplementary Figure 2. AFM analysis reveals β$_2$m variant fibril handedness and height distributions.

a, Examples of fibrils from each β$_2$m sample imaged by AFM, all of which demonstrate left-handed twists. Images display the changes in z-height across the sample surface. Handedness can be determined by observing that, for an AFM image of a given fibril, when rotated so that the fibril axis is horizontal, the peaks and valleys of the twist begin in the bottom left and move to the top right for left-handed fibrils and from bottom right to top left for right-handed fibrils. All scalebars represent 10 nm. **b**, Measured fibril height distributions from AFM images of the three β$_2$m variants, for n number of fibrils. Heights corresponding to fibril width ranges measured from cryoEM 2D class averages of the solved fibril forms are annotated onto the charts as coloured bands. For β$_2$m-V27M, the 1PF and 2PF bands are indicative of the predicted positions for these fibril types if similar species are present to those found in the β$_2$m-D76N and β$_2$m-ΔN6 datasets respectively. Source data are provided as a Source Data file.

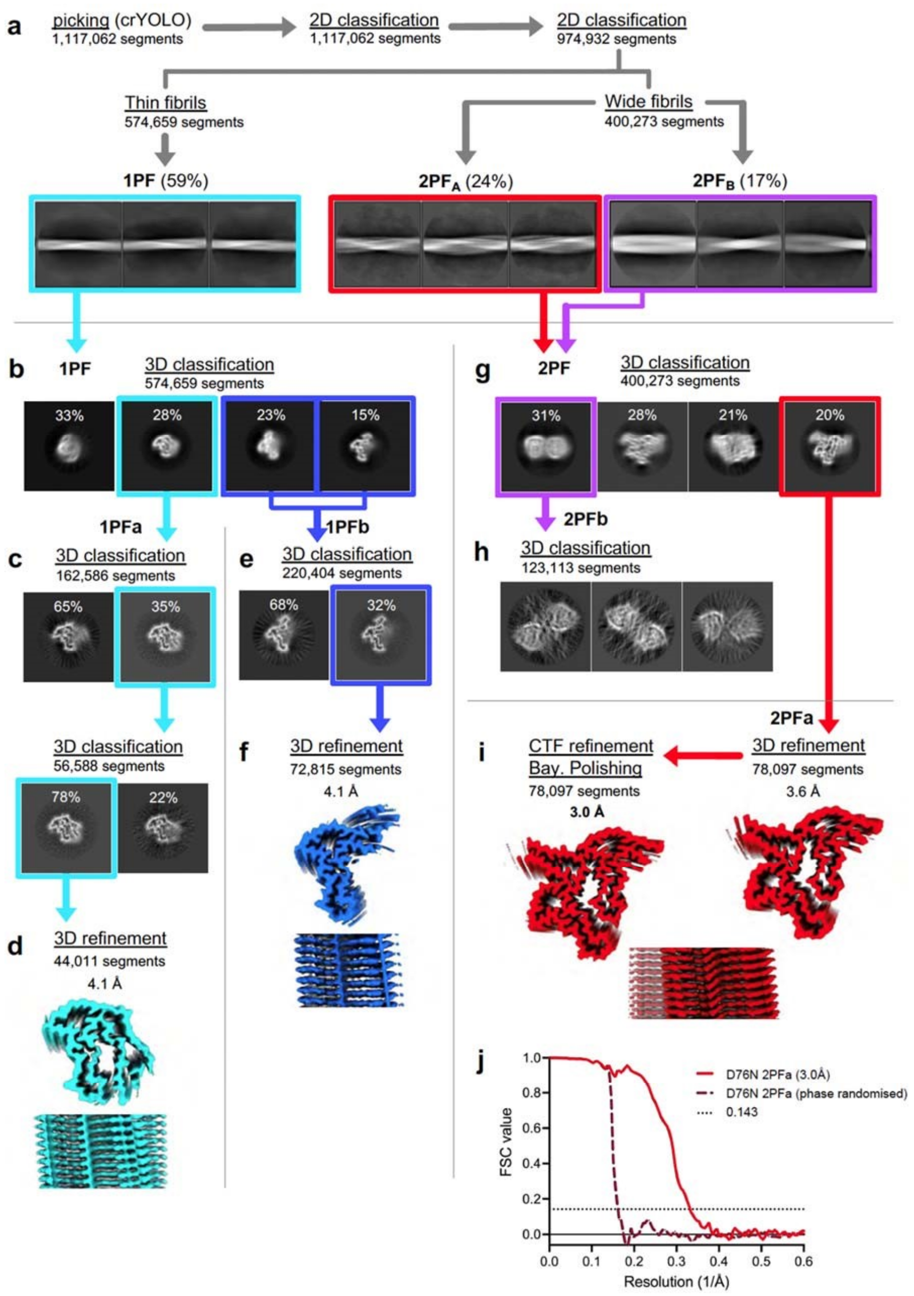

Supplementary Figure 3. Processing summary for the β_2m-D76N cryoEM dataset.

a, Summary of the initial processing steps for the β_2m-D76N cryo-EM dataset. After multiple rounds of cleaning by 2D classification, the dataset was split into two based on the width of the fibril segments, with example 2D class averages from 2x binned segments displayed for each observed form. **b**, 3D classification of the major subset containing 1PF fibrils revealed different positions for the two protein termini, both facing either down (1PFa) or up (1PFb). **c**, **e**, The 1PFa and 1PFb subsets were further processed separately. **d**, **f**, After 3D refinement the separation of helical layers could not be completely resolved for either 1PFa or 1PFb, and so we did not consider the structure solved, and maps and models were not deposited. **g**, 3D classification of the 2PF segments led to the separation of the 2PFa and 2PFb fibril forms. **h**, The 2PFb fibril structure could not be resolved beyond showing two neighbouring blobs. **i**, The final 2PFa map was solved to 3.0 Å resolution, displayed with a sharpening value of -30 Å^2. **j**, FSC plots of the solved β_2m-D76N 2PFa fibril structure with the corresponding phase randomised FSC curve as a dashed line. Source data are provided as a Source Data file.

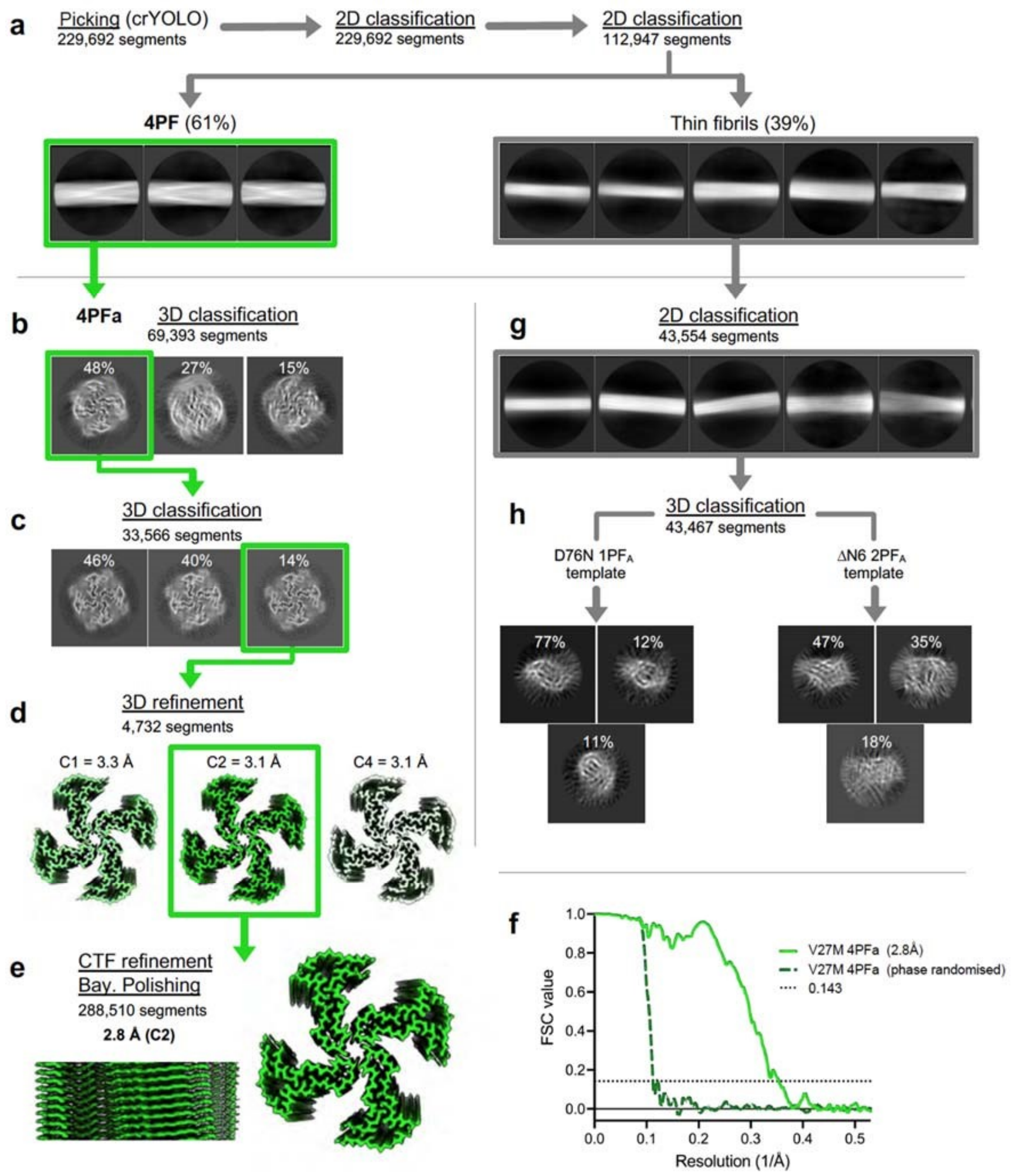

Supplementary Figure 4. Processing summary for the β$_2$m-V27M cryo-EM dataset

a, Summary of the initial processing steps for the β$_2$m-V27M cryo-EM dataset. After multiple rounds of cleaning by 2D classification, the dataset was split into two based on the width of the fibril segments, with example 2D class averages from 2x binned segments displayed for each observed form. **b**, **c**, The major 4PFa segments were processed as shown. **d**, The resulting 4PFa maps are displayed after refinement with either C1, C2 or C4 symmetry applied. The C4 map showed distortions in the backbone density at the outer regions of the fibril core so C2 was selected. **e**, The final 4PFa map was solved with C2 symmetry applied to 2.8 Å resolution, displayed with a sharpening value of -38 Å^2. **f**, FSC plots of the solved β$_2$m-V27M 4PFa fibril structure with the corresponding phase randomised FSC curve as dashed lines. **g**, Further 2D classification of the thin fibril segments did not reveal many internal features of the fibrils. **h**, 3D classification using either the solved D76N 1PFa or the ΔN6 2PFa fibril structure as starting templates did not reveal any solvable thin fibril forms from the data.

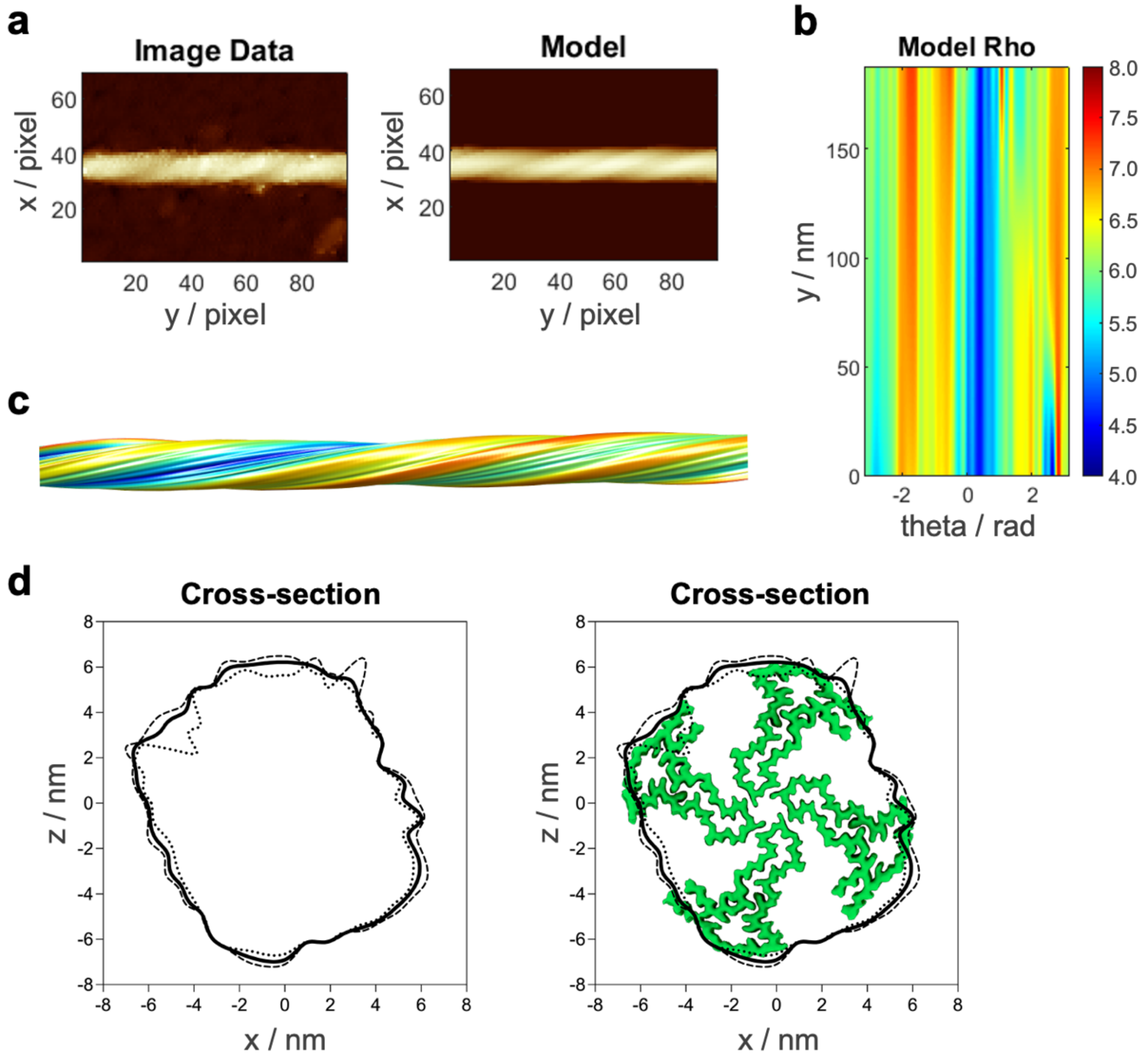

Supplementary Figure 5. Reconstruction of an AFM cross-section of a wide β₂m-V27M fibril resembles the 4PF fibril structure.

a, Images of the fibril surface for a selected β_2m-V27M wide fibril from an AFM image (*left*) and a corresponding computational model of the fibril generated using TraceY rendered to appear how it would look in AFM images[69] (*right*). **b**, Model Rho plot showing positions along the surface of the computational β_2m-V27M wide fibril model along the fibril axis (y) rotated around theta with their relative heights from the centre of the fibril cross-section (nm) coloured according to the scale. **c**, Side view of the reconstructed wide fibril, coloured by the relative surface height from the centre of the fibril as in **b**. **d**, Cross-section of the reconstructed AFM model shown alone (*left*) and with the scaled cryoEM map of the V27M 4PF fibril superposed (*right*) showing very similar cross-section dimensions. Source data are provided as a Source Data file.

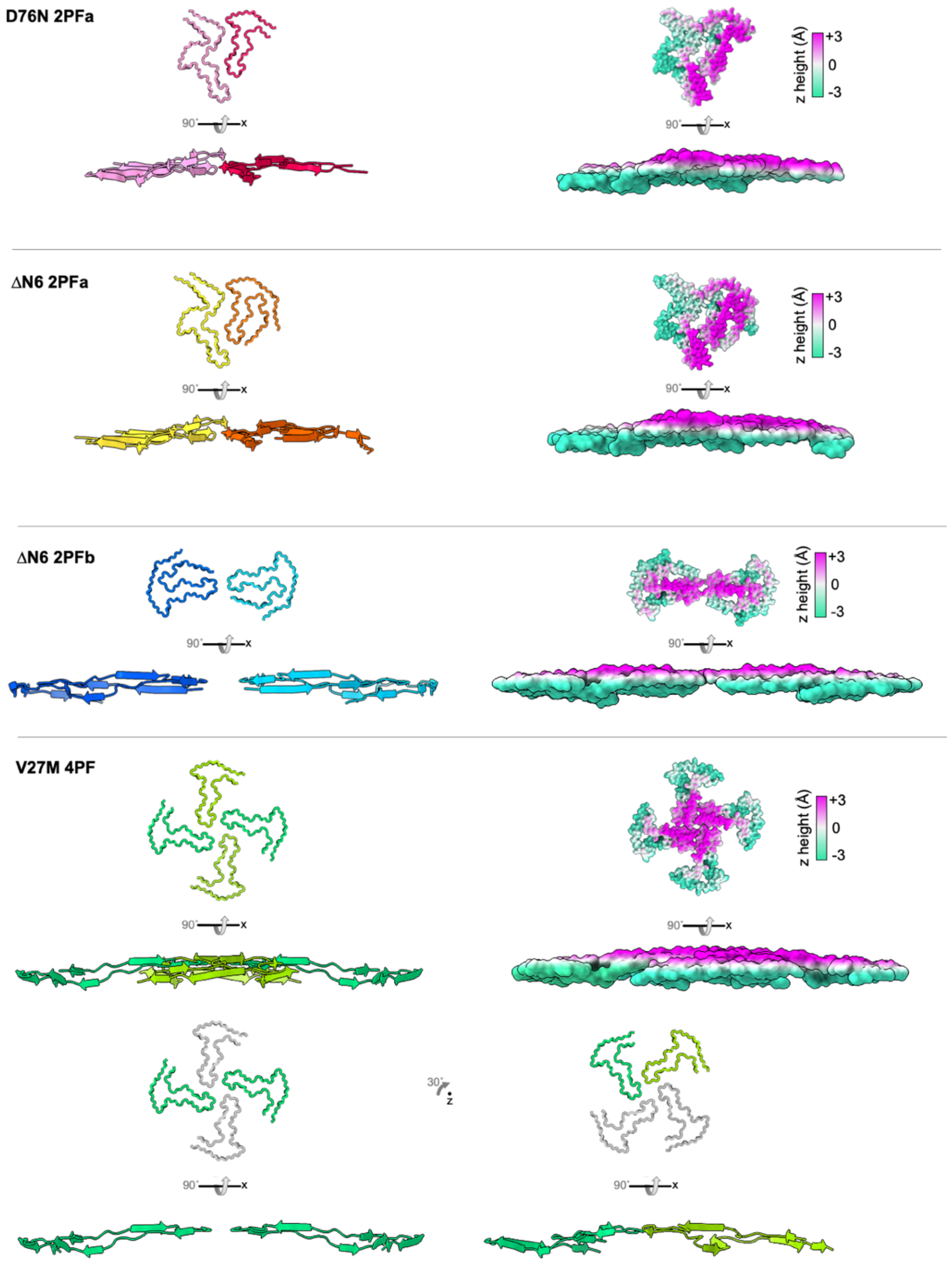

Supplementary Figure 6. Each of the β_2m variant fibril structures shows a common kinked subunit planarity. For each model, perpendicular views of the cartoon (*left*) and surface-filled (*right*) model for the base repeating helical unit of the respective fibril structure is shown. The surface model is coloured by relative height along the fibril z-axis as indicated by the scale bars. All of the subunits within the different fibril types have similar kinked planarity as shown by their similar pattern of height profiles across the subunit fold. For β_2m-V27M fibrils, an extra line of cartoon models is shown to highlight the relative orientations of different pairs of subunits within the 4PF core.

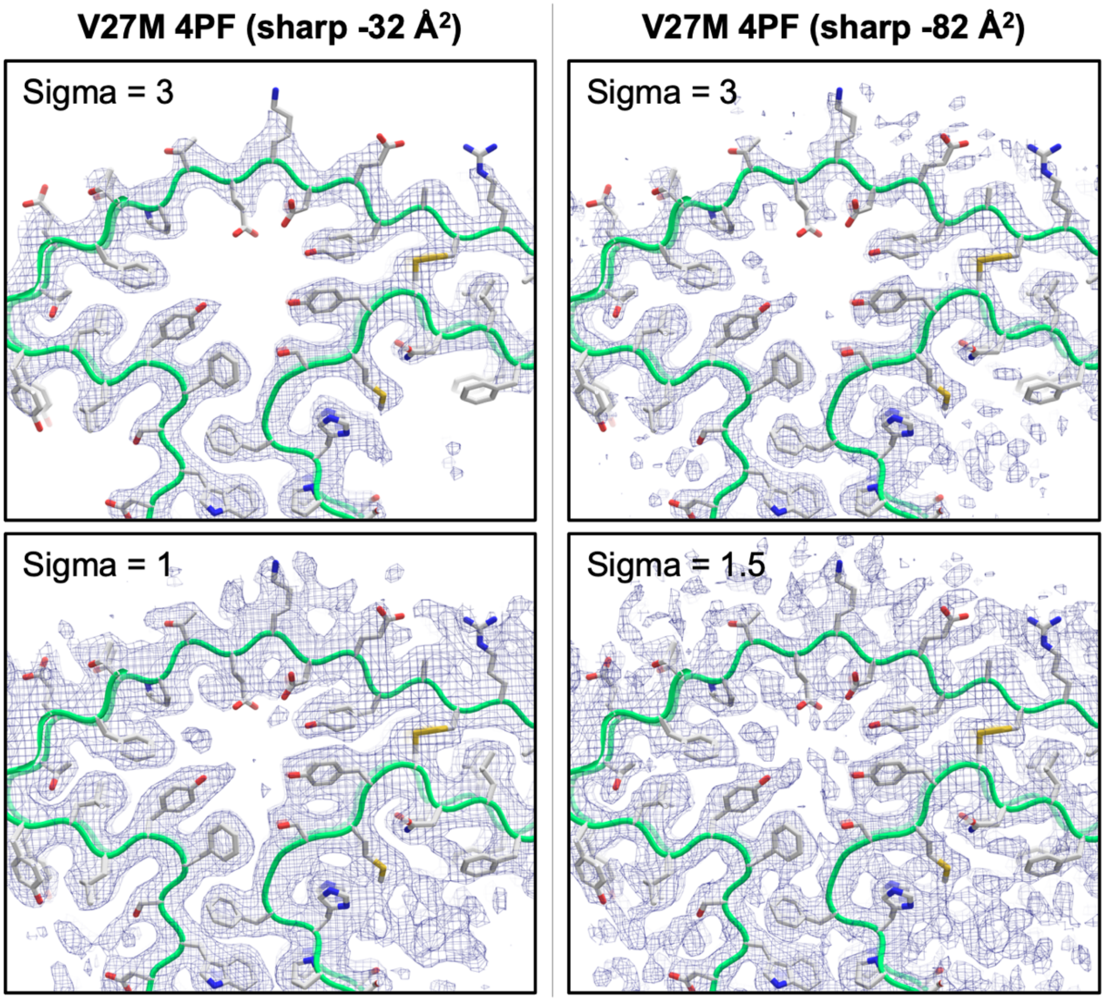

Supplementary Figure 7. Different displays of the highest resolution variant map suggests that the hammer-head pocket is filled by solvent
Overlay of the β_2m-V27M model with the associated EM density map showing the head of the hammer fold in one of the protofilaments of the 4PF fibril structure. The different panels represent different displays of the map, the left-side shows the map with the sharpening value used for deposition to the EMDB (and other figures in this manuscript) and the right-side shows the map with additional sharpening to try to elevate the signal within the pocket. The upper and bottom panels represent different display sigma contouring of each map. In all, there is not the resolution in the data to confirm what occupies the hammer-head pocket, however from these images it appears most likely to be filled with partially ordered solvent molecules.

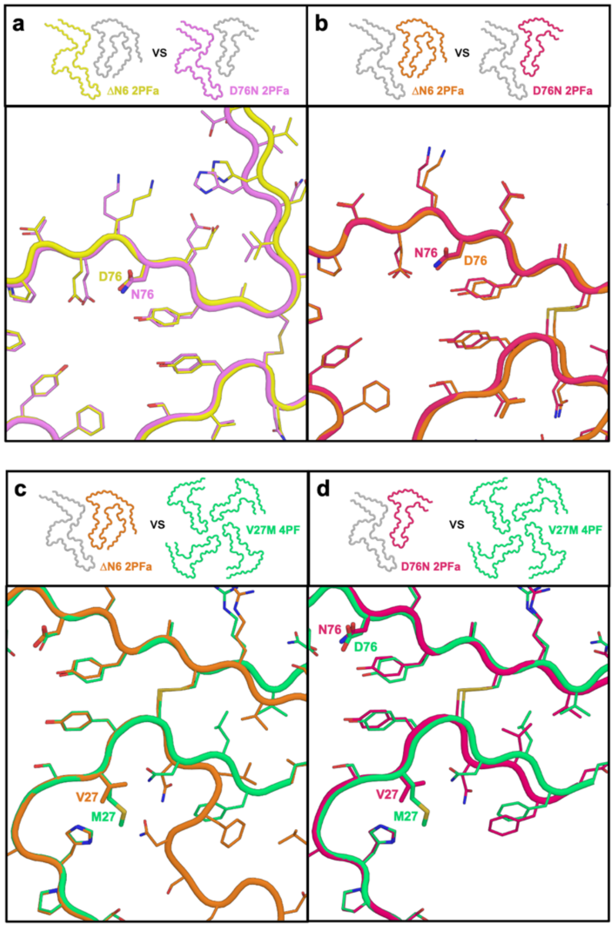

Supplementary Figure 8. Overview of the local structural alterations induced by the D76N and V27M mutations.

Section of a superposition of the ΔN6-2PFa and D76N-2PFa fibril models aligned on residues 25-75 for a, protofilament 1 and b, protofilament 2 of each model respectively. D76/N76 is shown in sticks with all other side chains shown as lines. In both protofilaments there are minor changes around D76N within the hammer but these are insignificant in relation to the resolution of the determined structures. In protofilament 1 there is a significant movement of the termini b-strands beyond the disulphide bridge, which are positioned up to 3 Å closer to the hammer-head in the D76N structure. c, d, Similar graphics to a, and b, showing a superposition of one of the identical V27M subunits with protofilament 2 of ΔN6-2PFa and D76N-2PFa fibrils respectively, sectioned around the V27M mutation. No significant alterations are seen within the residue 25-80 fold enclosed by the disulphide bridge, but again adjustments are seen in the termini. The V27M mutation appears to create a steric clash to N21 in the ΔN6 conformation and in the V27M structure the termini occupy a position in the middle of the termini up and the termini down conformations. It is possible (albeit not conclusive) that this mutation and potential steric block could directly prevent the termini down conformation and cause the novel propellor-like 4PF arrangement only seen in the β2m-V27M fibril population.

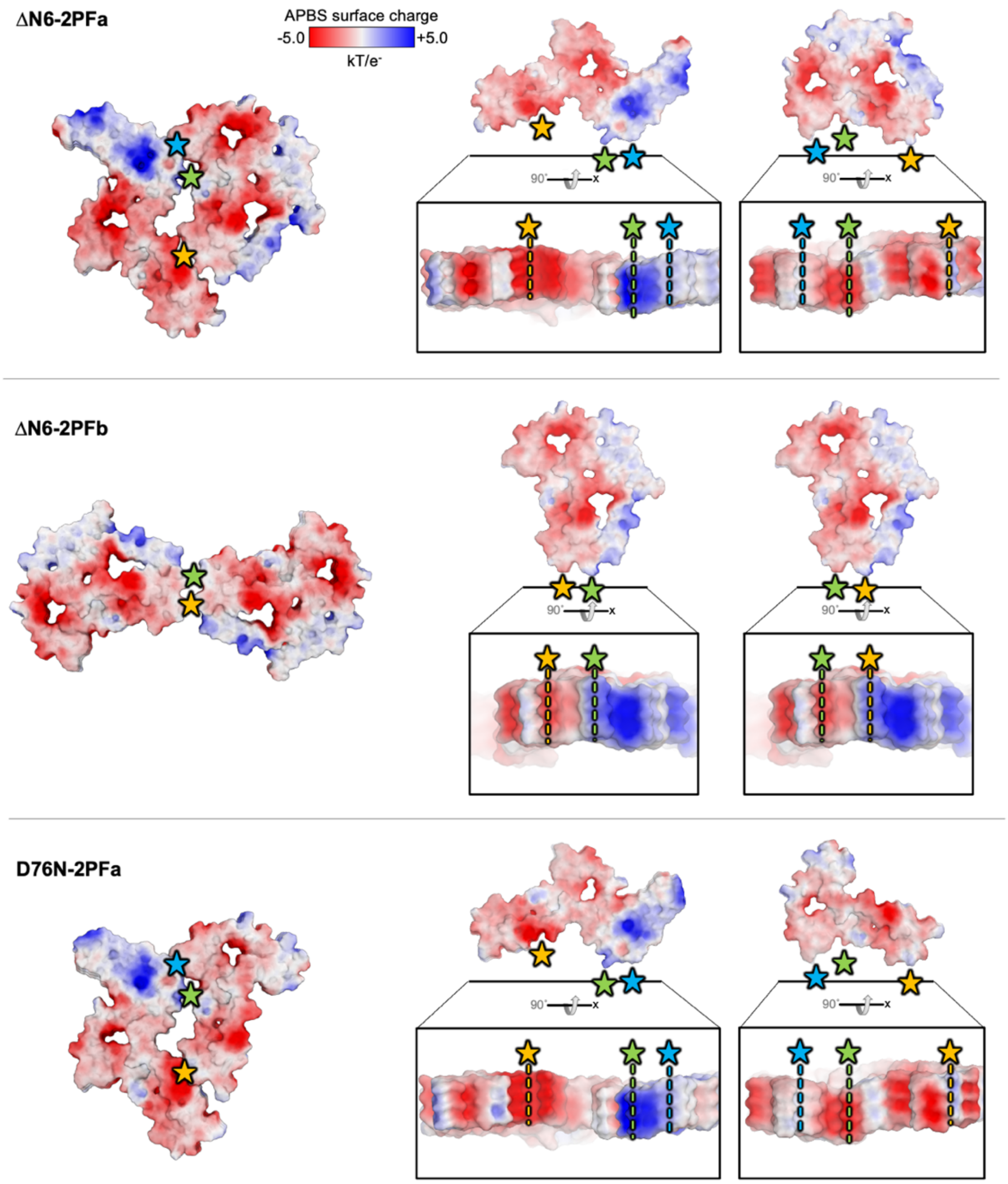

Supplementary Figure 9. Analysis of the fibril surface charge and its influence on inter-protofilament interactions for the β$_2$m-ΔN6 and β$_2$m-D76N fibril structures.

APBS electrostatic-coloured surfaces (generated using PyMol) of each fibril type with inter-protofilament interaction surfaces highlighted with coloured stars. A top-view of each fibril end surface (*left*) shows the location of the interaction sites. Each separate protofilament is shown (*right*) orientated to place the interaction surface facing the bottom of the page. Below each of these is a perpendicular view of the grooves along the fibril axis with the interacting regions again highlighted is complementary colours (i.e., the yellow site of one protofilament interacts with the yellow site on the other protofilament).

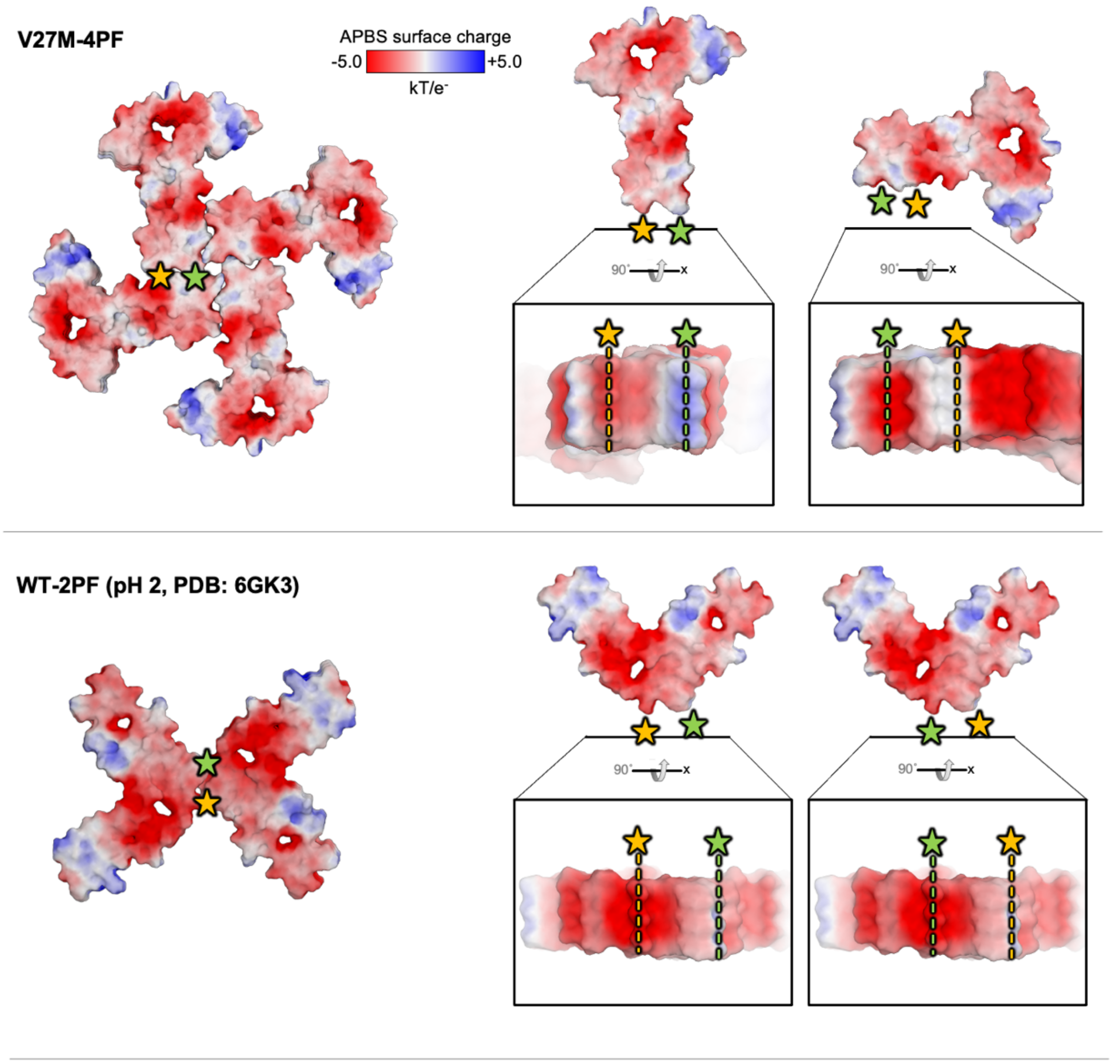

Supplementary Figure 10. Analysis of the fibril surface charge and its influence on inter-protofilament interactions for the β$_2$m-V27M and WT-β$_2$m acidic pH fibril structures.

APBS electrostatic-coloured surfaces of each fibril type with inter-protofilament interaction surfaces highlighted with coloured stars as described in **Extended Data Fig. 9**.

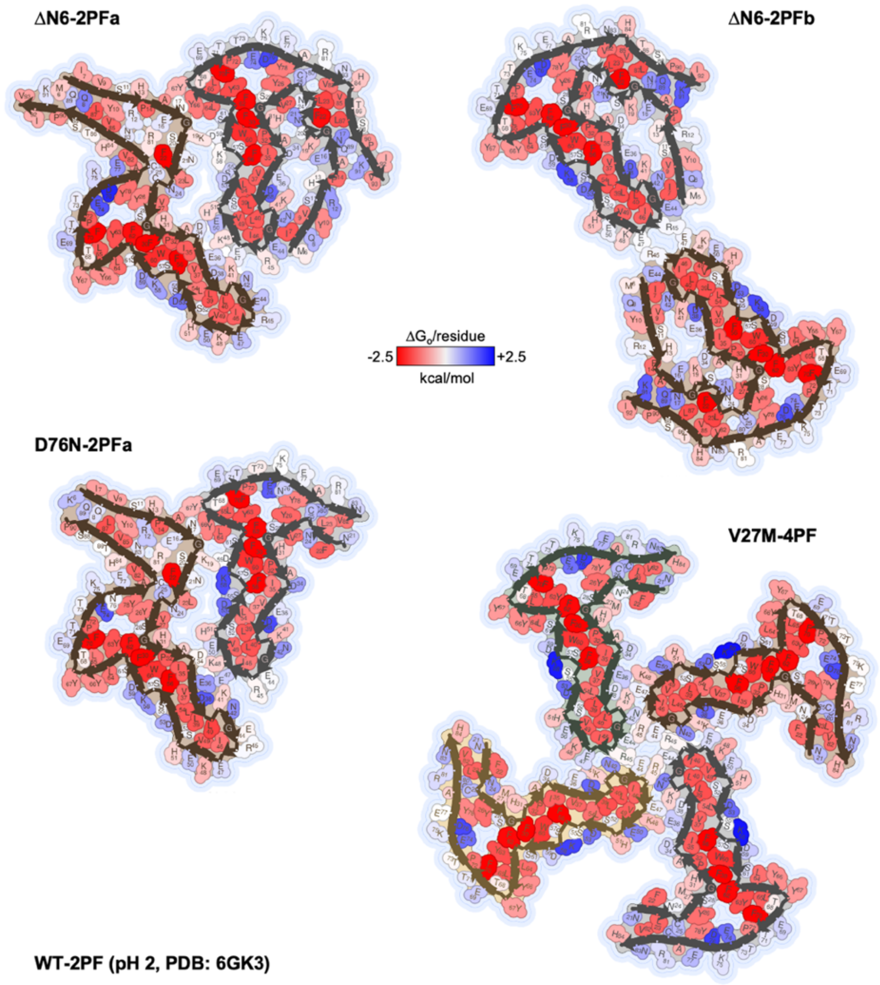

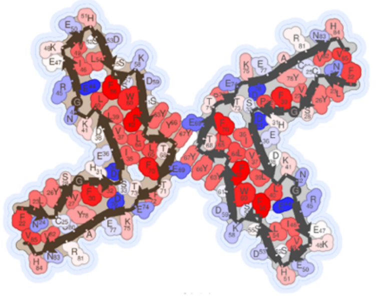

Structure	PFs	ΔGo/layer (kcal/mol)	ΔGo/residue (kcal/mol)
ΔN6-2PFa	2	-85.8	-0.49
ΔN6-2PFb	2	-82.8	-0.48
D76N-2PFa	2	-70.8	-0.48
V27M-4PF	4	-117.6	-0.46
WT (pH 2)	2	-67.0	-0.52

Supplementary Figure 11. Thermodynamic stability calculations for each β$_2$m fibril structure.
Outputs from thermodynamic stability calculations as used for Amyloid Atlas[12] using scripts kindly provided by Michael Sawaya. One fibril layer of each structure is shown with surface boundaries for each amino acid side chain coloured red-to-blue based on the calculated per residue ΔG° contribution towards the fold stability, as indicated by the colour scale. A summary table is shown detailing the overall ΔG° for one fibril layer (as shown in the molecule displays) and the average per residue ΔG° for each structure. This highlights that whilst the different structures have different apparent stabilities, they all actually have similar stabilities per residue when you take the number of residues ordered in each fibril structure into account.

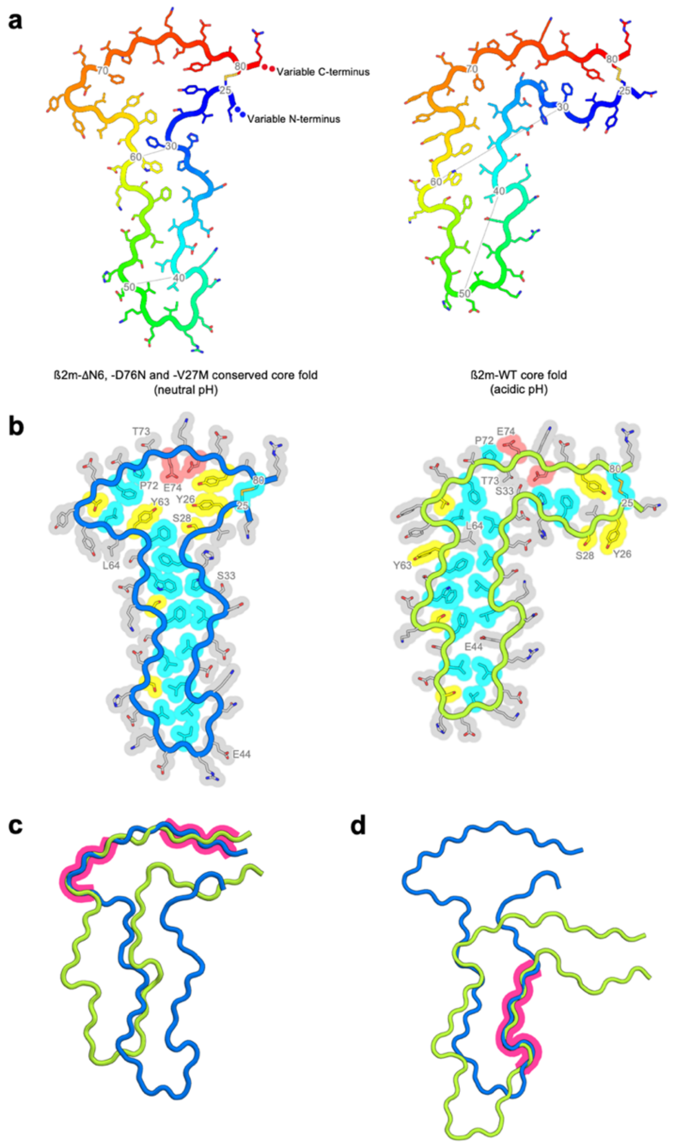

Supplementary Figure 12. Comparison of the β_2m variant fold with the WT acidic pH structure.
The conserved hammer motif found in the β_2m-ΔN6, β_2m-D76N and β_2m-V27M fibril structures described in this study alongside the structure of the same region (residues 25-80) in the pH 2.5 WT-β_2m structure[29] (PDB: 6GK3). The pairwise RMSD between C_α atoms in ΔN6-2PFa and 6GK3 is 11.2Å. **a**, The models are coloured blue-to-red from N-to-C terminus with strings added to highlight the drastic changes in intramolecular distance between positions 30-60 and 40-50 in each fold. **b**, The models are shown with ambient outlines of each side-chain coloured based on their location in the β_2m-ΔN6, β_2m-D76N and β_2m-V27M fibril fold, with solvent exposed side chains grey and internal, buried side chains coloured by side chain chemical property (polar in yellow, hydrophobic in cyan and charged in red or blue respectively). Residues that differ in intra-subunit location between the models are labelled. **c**, **d**, Superpositions of cartoon-loop models of the variant fold and the WT acidic pH fold, aligned based on the backbone of the pink highlighted residues (the same residues in each fold) in each superposition. In all, these figures highlight the extent of the difference in sequence register and structural packing between the two folds.

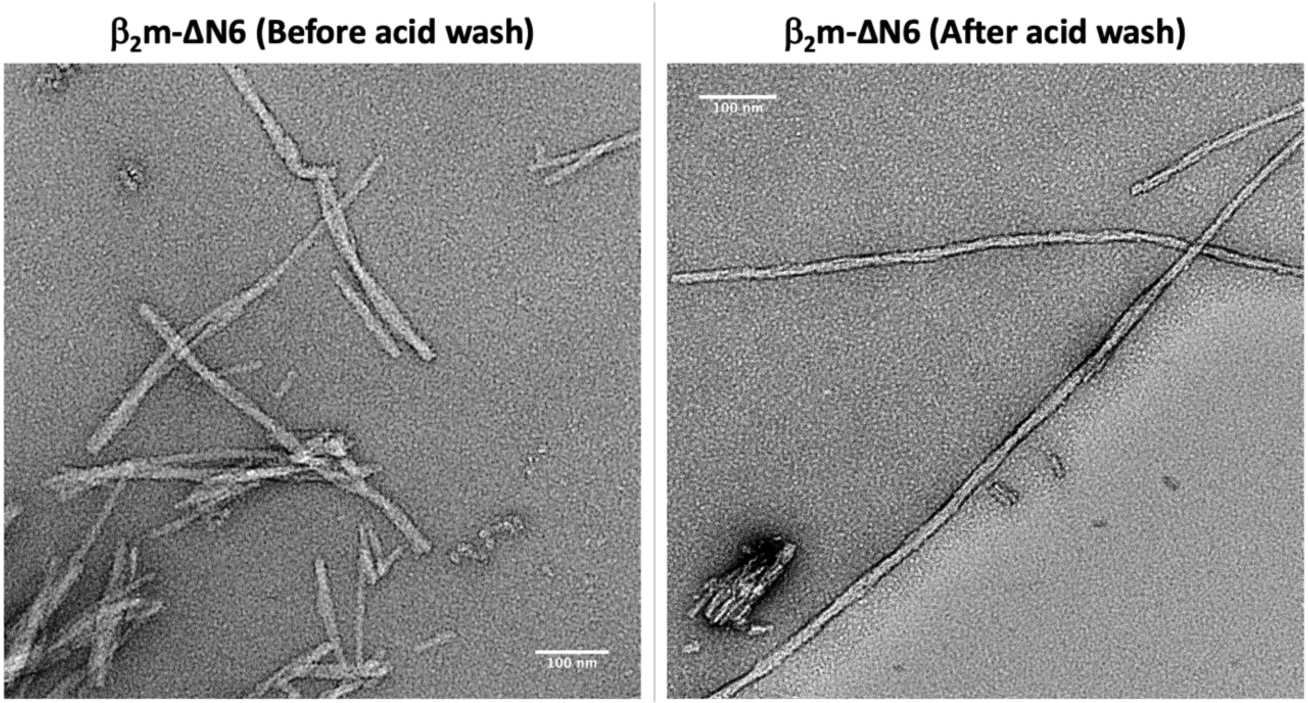

Supplementary Figure 13. A brief acid wash reveals fibrils suitable for structure determination.

Representative negative stain EM images (50,000x magnification) of β_2m-ΔN6 fibrils before and after a brief acid wash of pelleted material. Three independent experiments were performed, with similar results. CryoEM structures of the β_2m variants could not be solved until this step was implemented, as it reduced fibril tangling and cleared a fuzzy coat that hindered crossover identification.

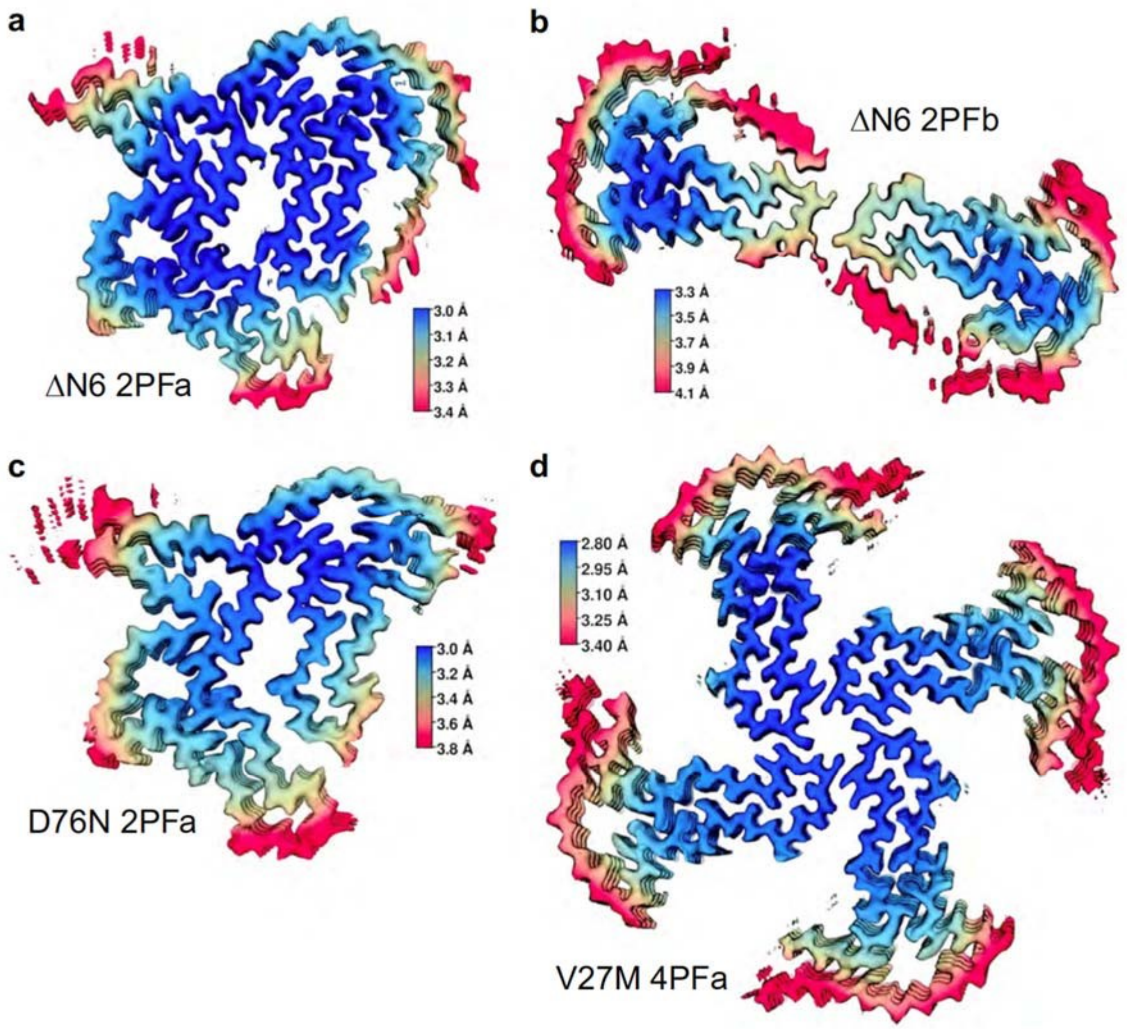

Supplementary Figure 14. Local resolution of the cryo-EM maps.

Local resolution-coloured maps of the the four fibril polymorphs solved to better than 4.0 Å resolution, which include: **a**, $β_2$m-ΔN6-2PFa, **b**, $β_2$m-ΔN6-2PFb, **c**, $β_2$m-D76N-2PFa and **d**, $β_2$m-V27M-4PFa. All local resolutions as calculated in RELION4[60].
